# Pan-cancer analysis of cuproptosis regulation patterns and identification of mTOR-target responder in clear cell renal cell carcinoma

**DOI:** 10.1186/s13062-022-00340-y

**Published:** 2022-10-08

**Authors:** Shichao Long, Ya Wang, Yuqiao Chen, Tianshu Fang, Yuanbing Yao, Kai Fu

**Affiliations:** 1 Institute of Molecular Precision Medicine and Hunan Key Laboratory of Molecular Precision Medicine, Department of General Surgery, Xiangya Hospital, Central South University, Changsha, Hunan China; 2 Center for Medical Genetics and Hunan Key Laboratory of Medical Genetics, School of Life Sciences, Central South University, Changsha, Hunan China; 3 Hunan Key Laboratory of Animal Models for Human Diseases, Central South University, Changsha, Hunan China; 4 National Clinical Research Center for Geriatric Disorders, Changsha, Hunan China; 5 Hunan Key Laboratory of Aging Biology, Xiangya Hospital, Central South University, Changsha, Hunan China

**Keywords:** Copper, Prognosis, Pan-cancer, Cuproptosis, mTOR

## Abstract

**Background:**

The mechanism of cuproptosis, a novel copper-induced cell death by regulating tricarboxylic acid cycle (TCA)-related genes, has been reported to regulate oxidative phosphorylation system (OXPHOS) in cancers and can be regarded as potential therapeutic strategies in cancer; however, the characteristics of cuproptosis in pan-cancer have not been elucidated.

**Methods:**

The multi-omics data of The Cancer Genome Atlas were used to evaluate the cuproptosis-associated characteristics across 32 tumor types. A cuproptosis enrichment score (CEScore) was established using a single sample gene enrichment analysis (ssGSEA) in pan-cancer. Spearman correlation analysis was used to identify pathway most associated with CEScore. Lasso-Cox regression was used to screen prognostic genes associated with OXPHOS and further construct a cuproptosis-related prognostic model in clear cell renal cell carcinoma (ccRCC).

**Results:**

We revealed that most cuproptosis-related genes (CRGs) were differentially expressed between tumors and normal tissues, and somatic copy number alterations contributed to their aberrant expression. We established a CEScore index to indicate cuproptosis status which was associated with prognosis in most cancers. The CEScore was negatively correlated with OXPHOS and significantly featured prognosis in ccRCC. The ccRCC patients with high-risk scores show worse survival outcomes and bad clinical benefits of Everolimus (mTOR inhibitor).

**Conclusions:**

Our findings indicate the importance of abnormal CRGs expression in cancers. In addition, identified several prognostic CRGs as potential markers for prognostic distinction and drug response in the specific tumor. These results accelerate the understanding of copper-induced death in tumor progression and provide cuproptosis-associated novel therapeutic strategies.

**Supplementary Information:**

The online version contains supplementary material available at 10.1186/s13062-022-00340-y.

## Introduction

Recently, Tsvetkov et al. identified and termed a copper-induced cell death (call ‘cuproptosis’) which distinct from ferroptosis, necroptosis and apoptosis [1]. Meanwhile, the pivotal mechanism of cuproptosis regulated via cuproptosis-related genes (CRGs) was also revealed. In the happened of cuproptosis, lipoyl moiety acts as a direct copper binder, causing lipoylated protein aggregation, Fe–S cluster–containing proteins lose, and the 70-kDa heat shock proteins elevation [2, 3]. In the past study, the copper chelator is an effective treatment for genetic abnormalities of copper homeostasis (such as Wilson's disease and Menke’s disease) [4]. Interestingly, dysregulated copper levels have been reported in patients suffering from differential cancers, including kidney, lung, breast, and colorectal cancers [3, 5–11]. Part of CRGs have been reported to play a significant role in tumors, including Ferredoxin 1 (FDX1) [12], pyruvate dehydrogenase E1 subunit alpha 1 (PDHA1) [13, 14], dihydrolipoamide dehydrogenase (DLD) [15, 16], and dihydrolipoamide S-acetyltransferase (DLAT) [17]. It indicates that targeting cuproptosis may be a potential therapeutic strategy in cancer. Although II-III clinical trials associated with copper ionophores in cancer have been conducted in recent years, the studies of target cuproptosis-associated molecules almost failed [18–21]. The main reason may be the failure to identify distinctive cuproptosis-related prognostic biomarkers and the selection of applicable cancer types. In addition, the cuproptosis characteristics based on multi-omics analyses haven’t been systemically clarified in cancers. Therefore, identifying cuproptosis patterns in pan-cancer may provide a novel insight for targeted therapy.

Hence, this study aims to assess the common or distinct cuproptosis status in tumors and to evaluate the appropriate therapeutic method. In this study, we comprehensively assessed the transcriptional and genomic features of CRGs among 32 solid tumors. Moreover, we characterized cuproptosis status based on CRGs expression and evaluated the correspondence between prognosis and cuproptosis. We found that cuproptosis significantly related to the cancer-associated pathway (especially oxidative phosphorylation) and overall survival rate in clear cell renal cell carcinoma (ccRCC). Subsequently, the constructed cuproptosis-related prognostic model shows precise discrimination in prognosis in ccRCC and the clinical benefit of mTOR inhibitor. Herein, we prove the crucial roles of cuproptosis in cancer.

## Methods

### Acquisition of data

The CRGs were extracted from the reports of Tsvetkov [1].The pan-cancer (32/32) normalized gene expression RNAseq data (Version: 2019.07.22; Platform: Illumina; Unit: Log_2_(fpkm-uq + 1); samples: 10,454), and corresponding clinical data were downloaded from the UCSC Xena website (https://xenabrowser.net/datapages/). Differential DNA methylation data of CRGs in TCGA pan-cancer (26/32) were acquired from the DNMIVD database (http://119.3.41.228/dnmivd/index/) [22]. Copy number variations of CRGs in TCGA pan-cancer (32/32) were extracted from the cBioPortal database. The somatic mutation profiles of CRGs of TCGA pan-cancer (32/32) based on the whole-exome sequencing platform were downloaded from cBioPortal [23].

The gene expression matrix, somatic mutations data, and relevant clinical files (E-MTAB-1980 and CheckMate-025) of validated ccRCC cohorts were downloaded from the ArrayExpress database (https://www.ebi.ac.uk/arrayexpress/experiments/E-MTAB-1980/) and supplementary information of Braun study, respectively [24]. The relevant expression array and clinical benefit information of Everolimus (mTOR inhibitor) in ccRCC were extracted from CheckMate-025 (CM-025).

### Differential expression analysis of CRGs

Firstly, we extracted 17 CRGs from Tsvetkov’s study [1]. To detect differential expression of CRGs between normal and tumor tissue in pan-cancer (23/32), the limma package was utilized to calculate the log_2_ fold change and adjusted *p*.value. Then, we define CRGs with an adjusted *p*.value < 0.05 and absolute Log_2_ fold change (|Log_2_FC|) value > 1 as cuproptosis-related differential expression genes.

### Somatic mutation and copy-number alteration (CNA) analysis

The maftools package was applied to import the samples with somatic CRGs mutation in TCGA pan-cancer (29/32) and CM-025 (patients with Everolimus treatment). The copy number alteration of each CRGs was evaluated for amplification and deletion. The mutation and CNA events of pan-cancer were integrated into the oncoplot of CRGs.

### Identification of prognostic genes in pan-cancer

The overall survival (OS) and progression-free survival (PFS) of patients in pan-cancer (32/32) based on the expression of CRGs were analyzed by GEPIA2 (http://gepia2.cancer-pku.cn/), which is an online web server for visualization of large-scale cancer-associated genomics’ expression profiles database [25].

### Calculate cuproptosis enrichment score (CEScore)

The GSVA (Gene Set Enrichment Analysis) package was utilized to calculate the CEScore using a single sample enrichment method [26]. These CRGs were considered as positive or negative factors of cuproptosis [1]. Therefore, 17 CRGs were enrolled into GSVA analysis to calculate the CEScore. The CEScore to illustrate the cuproptosis level was constructed based on the CRGs’ expression (including *FDX1, DLAT, DBT, DLD, GLS, PDHB, PDHA1, GCSH, CDKN2A, LIAS, ATP7A, LIPT2, ATP7B, LIPT1, SLC31A1, MTF1*, and *DLST*).

### Identified prognosis-related oncogenic genes based on CEScore

To screen oxidative phosphorylation genes significantly connected to CEScore, Spearman correlation analysis was conducted in pan-cancer (32/32). The screening condition is *p*.value < 0.05, and |R|≥ 0.7.

To identify the features of cuproptosis, the samples of pan-cancer (32/32) were separated into high-CEScore and low-CEScore (cutoff = median value of CEScore). Next, the hallmark gene sets (version: h.all.v7.5.1.symbols.gmt) was downloaded from MSigDB website (www.gsea-msigdb.org), and gene set enrichment analysis (GSEA) was performed using clusterProfiler package [27].

OncoScore, a text mining R package to assess the oncogenic potential of genes based on literature, was used to screen CEScore-related oncogenic genes in oxidative phosphorylation gene sets. The candidate genes of OncoScore > 21.7 were chosen to subsequently analysis [28]. OncoScape, an algorithm to identify new candidate cancer genes by using multi-omics data, was also utilized to screen oncogenic potential genes [29]. The Combined genes with OncoScape (OG score > 2 and different expression > 0) and OncoScore greater than 21.7 were included in the subsequent univariate Cox regression analysis.

### Prognostic risk signature construction in KIRC

Firstly, univariate Cox regression analysis was performed to screen prognostic genes associated with OS. All eligible genes (*p*.value < 0.05) were further included in Lasso analysis for dimension reduction using the glmnet R package. Then, 17 prognostic genes, as risk factors, were identified and utilized to construct the cuproptosis-related prognostic (CRP) model in KIRC. CRP scores of each sample in ccRCC were calculated in a linear combination of regression coefficient values and risk gene expression level. According to the median value of CRP scores, patients in KIRC are divided into the high- and low-risk groups.

### Construction and evaluation of nomogram

To construct a cuproptosis-related prognosis (CRP) model, the clinical prognosis factors were modeled by uni-, and multi-variate Cox risk regression in the TCGA-KIRC cohort. The clinical characteristics were transformed into binary variables, including Age (< = 65: 1, > 65: 2), Gender (Male: 1, Female: 2), Stage (Stage I–II: 1, Stage III–IV: 2), Grade (G1–G2: 1, G3–G4: 2).

The Nomograms were constructed to predict OS and PFS probabilities specify years (1, 3, and 5) by integrating clinical data. And calibration curves and decision curves analyses were performed to assess the accord between the predicted and actual OS and PFS rates via rms package.

### Statistical analysis

All statistical analyses of data were processed with R 4.1.0 software. Student's t-tests and Mann–Whitney U were executed to compare differences between the two groups. The chi-square or Fisher’s test was applied to compare proportional differences. Kaplan–Meier (KM) analysis and log-rank test were conducted to compare OS and PFS between the two groups.

## Result

### The landscape of cuproptosis in pan-cancer

We exploited the cuproptosis status of 730 normal tissues and 9724 tumor tissues from the TCGA database, including 32 cancer types in mRNA levels, methylation, copy number variation (CNV), and somatic cell mutations.

**Fig. 1:**
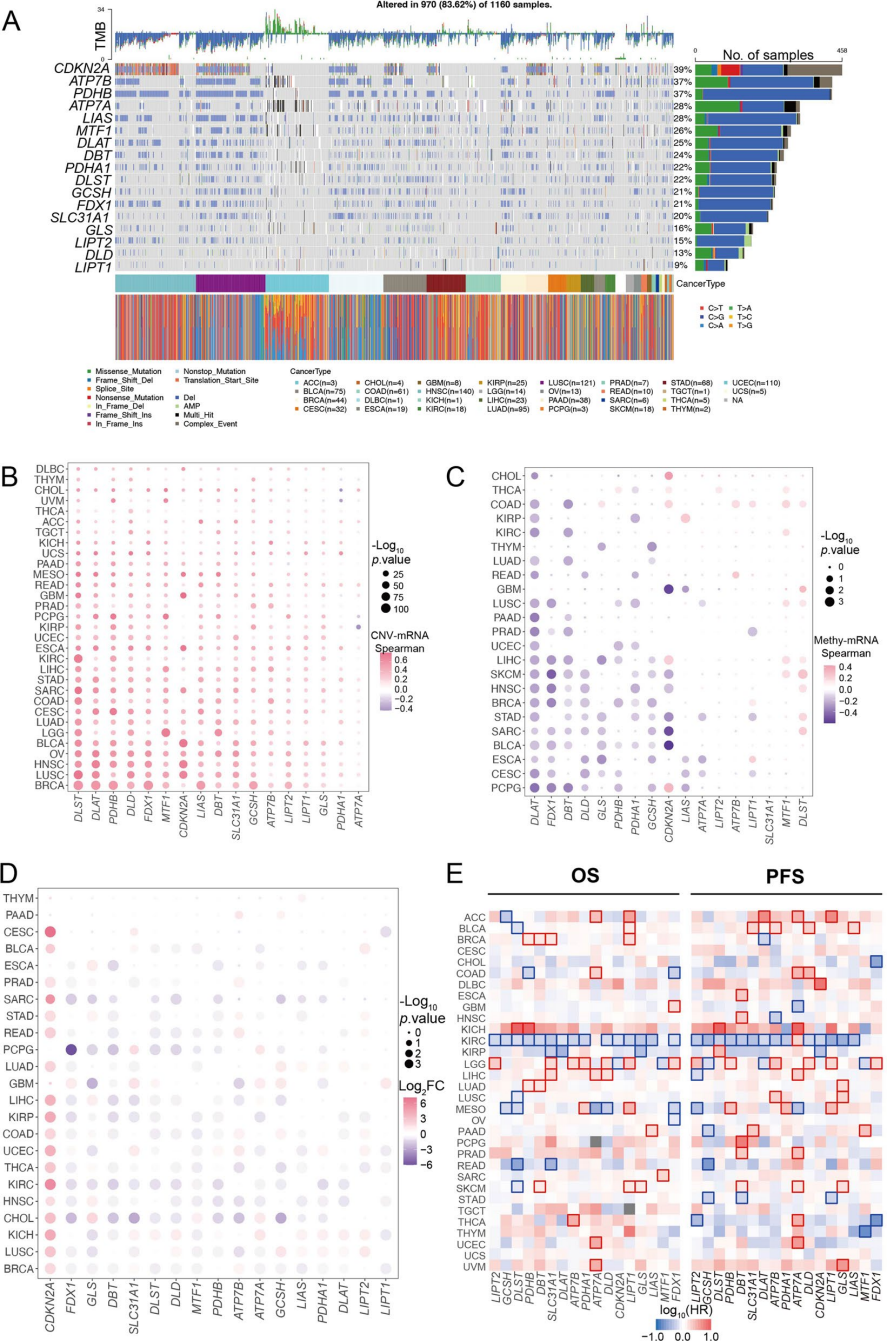
Distinct copper-regulated genes (CRGs) expression and modification characteristics and mutation-related analysis across 32 cancer types. **A** Somatic alteration in pan-cancer (29/32). Top histogram, tumor mutation rate; Bottom histogram, an indication of cohort and nucleotide mutation type; Right histograms, frequency of somatic alterations in each of CRGs. **B** Spearman’s correlation between transcriptional levels of CRGs and these genes’ promoter methylation; Significantly positive and negative correlation are marked in red and purple, respectively. **C** The Spearman’s correlation of CRGs between transcriptional level and somatic copy number alterations. **D** The heatmap shows differential expression fold change of CRGs in each cancer (23/32). The red and blue points are marked significantly up-, and down-regulated genes, respectively. **E** The overall survival (OS) and progression-free survival (PFS) of CRGs in 32 cancers. The risk and protective factor genes are marked red and blue, respectively. The border frame means significant differences in high- and low expression of genes

We firstly investigated the landscape of somatic mutation and CNV of CRGs in different cancers (29/32). *CDKN2A, PDHB, ATP7B, LIAS, ATP7A, and MTF1* have high mutation rates (Fig. 1A and Additional file 15: Table S2). There were more CRGs mutation and copy number *Del* in HNSC (n = 137, abbreviates see in Table 1), LUSC (n = 118), UCEC (n = 105), LUAD (n = 97), BLCA (n = 75), and STAD (n = 68). Furthermore, no somatic mutations of CRGs were detected in MESO (n = 86), and UVM (n = 80). In addition, almost all tumors were more prone to have copy number deletion than copy number amplification in CRGs, but COAD (n = 63) and UCEC (n = 547) showed the opposite profile. Interestingly, we found most mutational co-occurrence in pan-cancer, such as *DLAT* and *ATP7B* mutations, whereas less mutually exclusive mutation events were observed (Additional file 1: Fig. S1A).

**Table 1:** The number of tumors and normal tissues in the pan-cancer dataset

Abbreviation	Full name	Total	Tumor	Normal
ACC	Adrenocortical carcinoma	79	79	0
BLCA	Bladder Urothelial Carcinoma	430	411	19
BRCA	Breast invasive carcinoma	1210	1097	113
CESC	Cervical squamous cell carcinoma and endocervical adenocarcinoma	307	304	3
CHOL	Cholangiocarcinoma	45	36	9
COAD	Colon adenocarcinoma	510	469	41
DLBC	Lymphoid Neoplasm Diffuse Large B-cell Lymphoma	48	48	0
ESCA	Esophageal carcinoma	172	161	11
GBM	Glioblastoma multiforme	160	155	5
HNSC	Head and Neck squamous cell carcinoma	544	500	44
KICH	Kidney Chromophobe	89	65	24
KIRC	Kidney renal clear cell carcinoma	606	534	72
KIRP	Kidney renal papillary cell carcinoma	320	288	32
LGG	Brain Lower Grade Glioma	511	511	0
LIHC	Liver hepatocellular carcinoma	421	371	50
LUAD	Lung adenocarcinoma	583	524	59
LUSC	Lung squamous cell carcinoma	550	501	49
MESO	Mesothelioma	86	86	0
OV	Ovarian serous cystadenocarcinoma	374	374	0
PAAD	Pancreatic adenocarcinoma	181	177	4
PCPG	Pheochromocytoma and Paraganglioma	181	178	3
PRAD	Prostate adenocarcinoma	550	498	52
READ	Rectum adenocarcinoma	176	166	10
SARC	Sarcoma	261	259	2
SKCM	Skin Cutaneous Melanoma	104	103	1
STAD	Stomach adenocarcinoma	407	375	32
TGCT	Testicular Germ Cell Tumors	150	150	0
THCA	Thyroid carcinoma	560	502	58
THYM	Thymoma	121	119	2
UCEC	Uterine Corpus Endometrial Carcinoma	582	547	35
UCS	Uterine Carcinosarcoma	56	56	0
UVM	Uveal Melanoma	80	80	0

Since it has been proved that abnormal CNV can regulate gene expression, we explored the correlation between CNV levels and the mRNA expression of CRGs. The CNV levels of CRGs in most tumors were positively correlated with their mRNA levels, such as *DLST, PDHB, DLD, LIAS, DBT, ATP7B*. *LIPT1* (Fig. 1B and Additional file 16: Table S3). The results demonstrated that CNV levels can influence the mRNA expression of CRGs in most cancers. We also evaluated hyper- and hypo-methylation of the above CRGs between tumor and normal tissues. Although CRGs have less significantly differential methylation levels in cancers (Additional file 1: Fig. S1B), most mRNA levels of CRGs are negatively correlated with methylation levels in specific tumors (Fig. 1C). The transcriptional levels of *DLAT, FDX1, DBT, DLD, PDHB*, and *GCSH* negatively correlate with methylation in part of tumors (> 7 cancer types). In contrast, *MTF1* and *DLST* have a significantly positive correlation in some types of tumors. It means that abnormal methylation modification still affects CRG mRNA expression.

Subsequently, we evaluated the differential mRNA levels of *FDX1, LIAS, LIPT1, DLD, PDHA1, DLAT, PDHB, MTF1, GLS, CDKN2A, ATP7A, ATP7B, SLC31A1, DLST, DBT, GCSH*, and *LIPT2* in TCGA RNA-seq data (23/32). Most cancers show significantly lower mRNA levels in *FDX1, DLD, DLST, LIAS, GLS, DBT, MTF1*, and *PDHB*. In addition to the high expression of *CDKN2A* in tumor tissue, the rest of the genes showed low expression in tumors (Fig. 1D and Additional file 17: Table S4). Similarly differential gene expression analysis results also performing between paired tumor and normal samples in pan-cancer cohort (14/32, Additional file 2: Fig. S2A). Noticeable, most of CRGs were significantly differential expression in KIRC cohort.

In addition, to further reveal the clinical relevance of cuproptosis, the affection of CRGs in survival was decoded. The OS and progression-free survival (PFS) analyses demonstrated that most CRGs serve as a protective or risk factor for at least two cancer types (Fig. 1E). Almost all CRGs were cited as a significant protective factor in KIRC (n = 534).

The gene expression and cancer-associated subtype analysis are utilized to identify subtype relevant changes of gene expression. The results show that most of CRGs have significant differences between different subtypes in KIRC and BRCA (Additional file 2: Fig. S2B and Additional file 18: Table S5). Thus, diverse cuproptosis-regulated patterns in different cancers suggest that the genomic and transcriptome characteristics of CRGs were tumor-specific and the correlation between CRGs and prognosis deserves further study.

### Construction and characterization of CEScore in pan-cancer

**Fig. 2:**
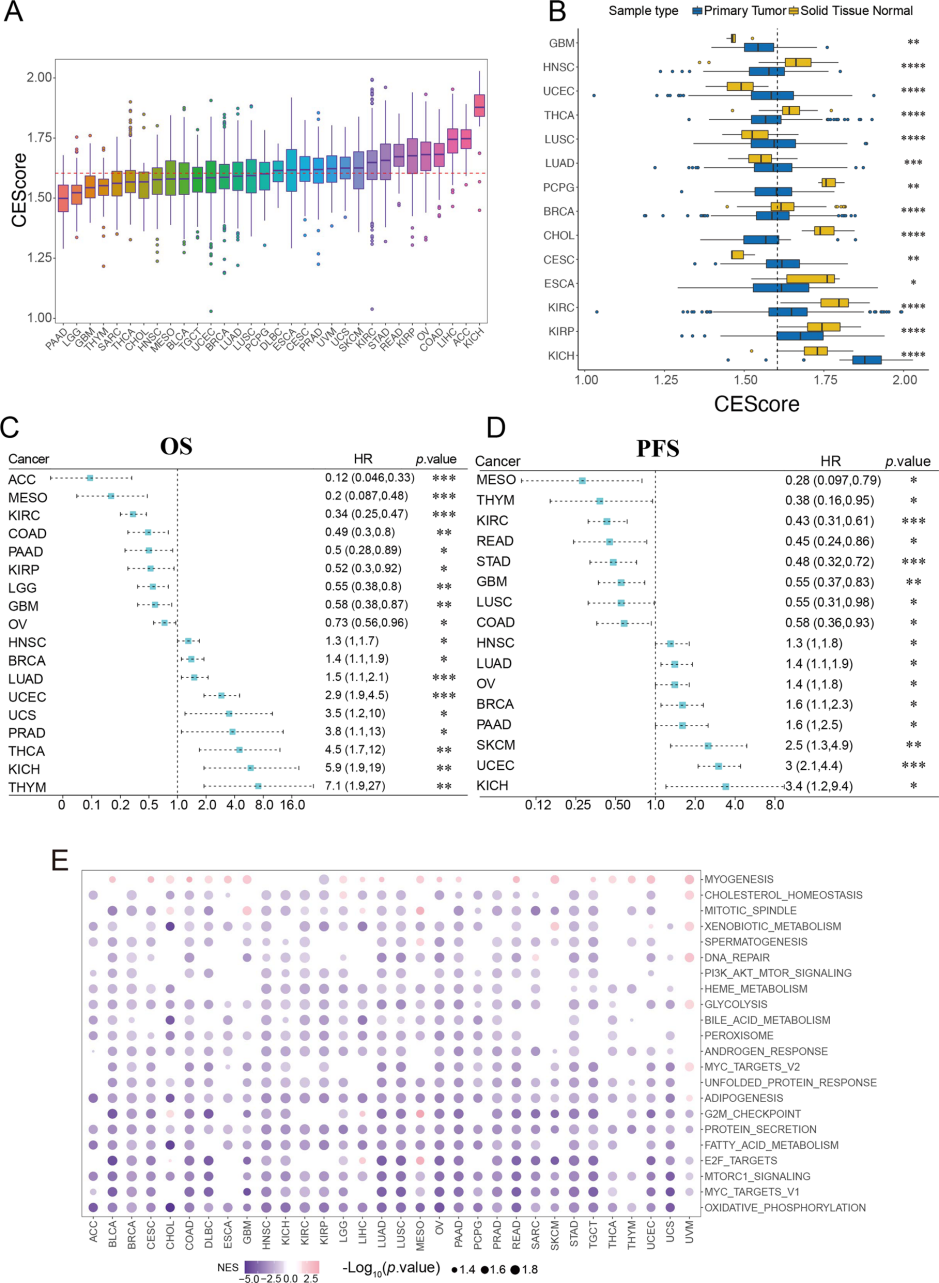
The characteristics of cuproptosis enrichment score (CEScore) among 32 cancers. **A** The differential levels of CEScore in 32 cancers. **B** The differential levels of CEScore between tumor and normal in cancers (20/32). **C**, **D** Kaplan–Meier (KM) survival analysis of overall and progress-free according to the CEScore among cancers. **E** Enrichment analysis of tumor-related pathways significantly associated with CEScore

To further dissect the relevant clinical status associated with cuproptosis, the CEScore was calculated by GSVA in the pan-cancer cohort. The distribution of CEScore was observed in 32 TCGA tumors, and among 32 cancers, the KICH (n = 65) owns the highest CEScore, while the PAAD (n = 177) owns the lowest CEScore (Fig. 2A and Additional file 19: Table S6). Interestingly, the CEScore of kidney-related tumors, including KICH (n = 65) and ACC (n = 79) were higher than the median CEScore. However, those patients with KIRC (n = 534) and KIRP (n = 288) have lower CEScore than the median CEScore (Fig. 2B). We assessed the correlation between the CEScore and the survival (OS and PFS) of patients in the pan-cancer dataset (32/32, Fig. 2C, D and Additional file 20: Table S7). Cox regression analysis revealed that the CEScore significantly correlated with OS in 18 types and PFS in 16 cancers. The OS results of pan-cancer (32/32) demonstrated that the relationship between CEScore and prognosis depended on specific tumor type (Additional file 3: Fig. S3). In addition, a similar effect also emerged in the KM PFS results of pan-cancer (Additional file 4: Fig. S4). Interestingly, the results obtained from Cox regression analysis of OS (*p* < 0.001) and PFS (*p* < 0.001) illustrated that CEScore is a significant prognostic factor in KIRC. Relative baseline metadata see in Additional file 21: Table S8.

To further dissect the characteristics of cuproptosis in cancers, we calculated the Spearman correlation between CEScore and the whole transcriptional gene panel. Subsequently, genes with high correlation were utilized to perform GSEA in pan-cancer. The top 22 significantly cancer-associated terms of the GSEA were identified (Fig. 2E), including oxidative phosphorylation, MYC target, the mTORC1 signaling, E2F target, metabolism-associated pathway, DNA repair, PI3K/AKT/mTOR signaling.

### Identification of CEScore-associated prognostic genes in ccRCC

**Fig. 3:**
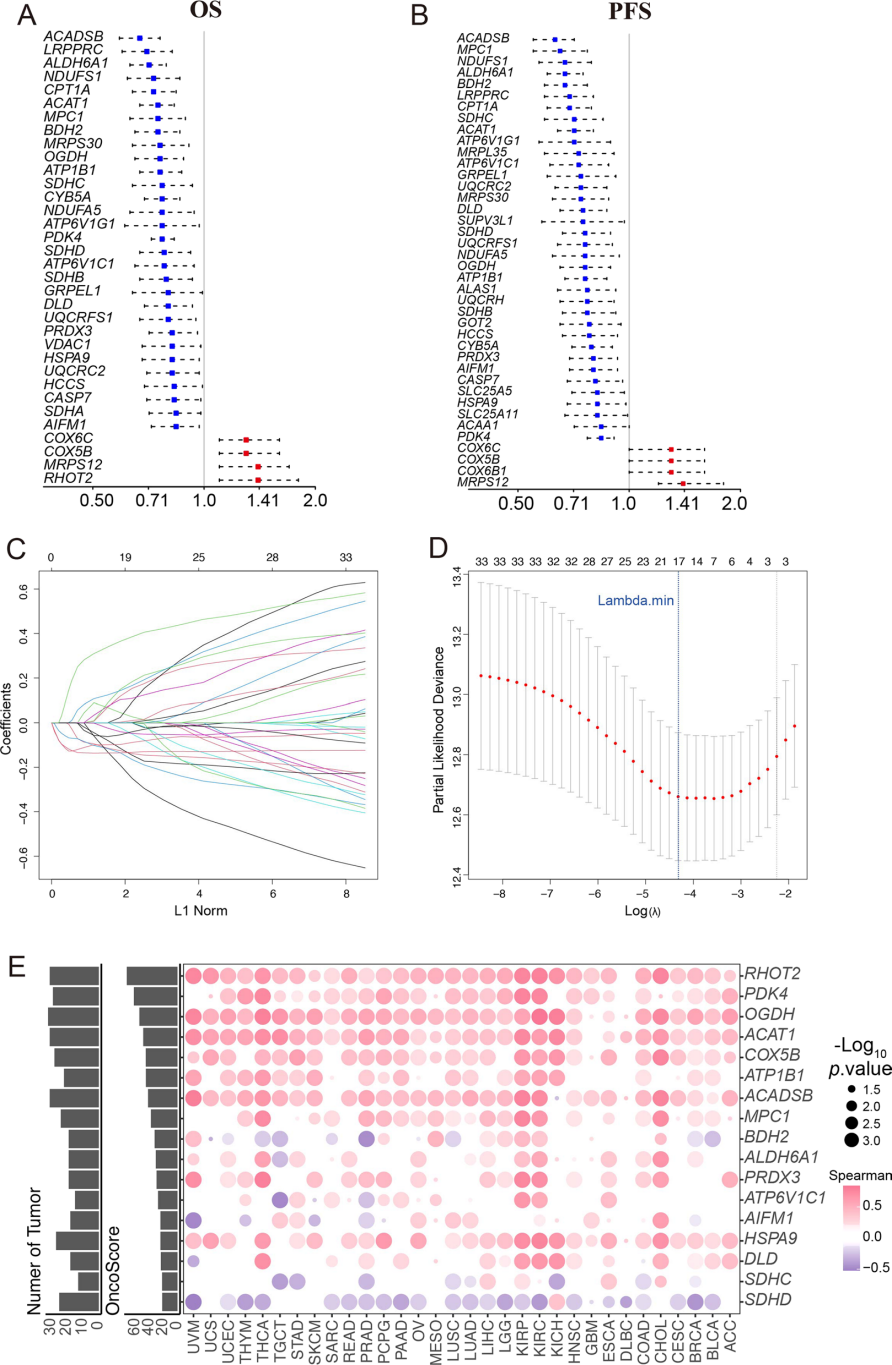
Exploration of CEScore-related oncogenic genes in TCGA-KIRC. (**A**, **B**) Univariate Cox regression analysis of the CEScore-related genes for OS (**A**) and progress free survival (PFS, **B**). **C**, **D** Lasso regression was utilized to screen optimal prognostic factors for OS in the TCGA-KIRC cohort (n = 353). Lasso coefficients panel of 32 variables (**C**) and 17 risk variables (**D**) were subsequently identified by lasso-Cox analysis for OS; Lambda.min: select the optimal risk score. **E** The candidate genes for cuproptosis-related prognostic (CRP) are significantly associated with CEScore in ≥ 10 cancer types. OncoScore: evaluate their oncogenic strength based on previous reports

The oxidative defense system is a characteristic of copper-associated deficiency disease [2, 30–34]. Hence, we extracted significant 181 CEScore-associated oxidative phosphorylation genes from the GSEA result (Additional file 22: Table S9). Firstly, we calculated the oncogenic potential genes using oncoScore and oncoScape algorithms in TCGA-KIRC (Additional file 23: Table S10). Then, the eighty-three candidate genes were included in univariate Cox regression analysis, and thirty-four OS-related genes and forty PFS-related genes were identified, respectively (Fig. 3A, B and Additional file 24: Table S11). Finally, seventeen significantly CEScore-associated prognostic genes (*RHOT2, PDK4, OGDH, ACAT1, COX5B, ATP1B1, ACADSB, MPC1, BDH2, ALDH6A1, PRDX3, ATP6V1C1, AFIM1, HSPA9, DLD, SDHC*, and *SDHD*) were identified by performing LASSO-Cox regression algorithm in KIRC (Fig. 3C, D). In addition, Spearman correlation analysis showed that 17 prognostic genes were significantly related to CEScore in pan-cancer (Fig. 3E).

### Construction and validation of CRP model in ccRCC

**Fig. 4:**
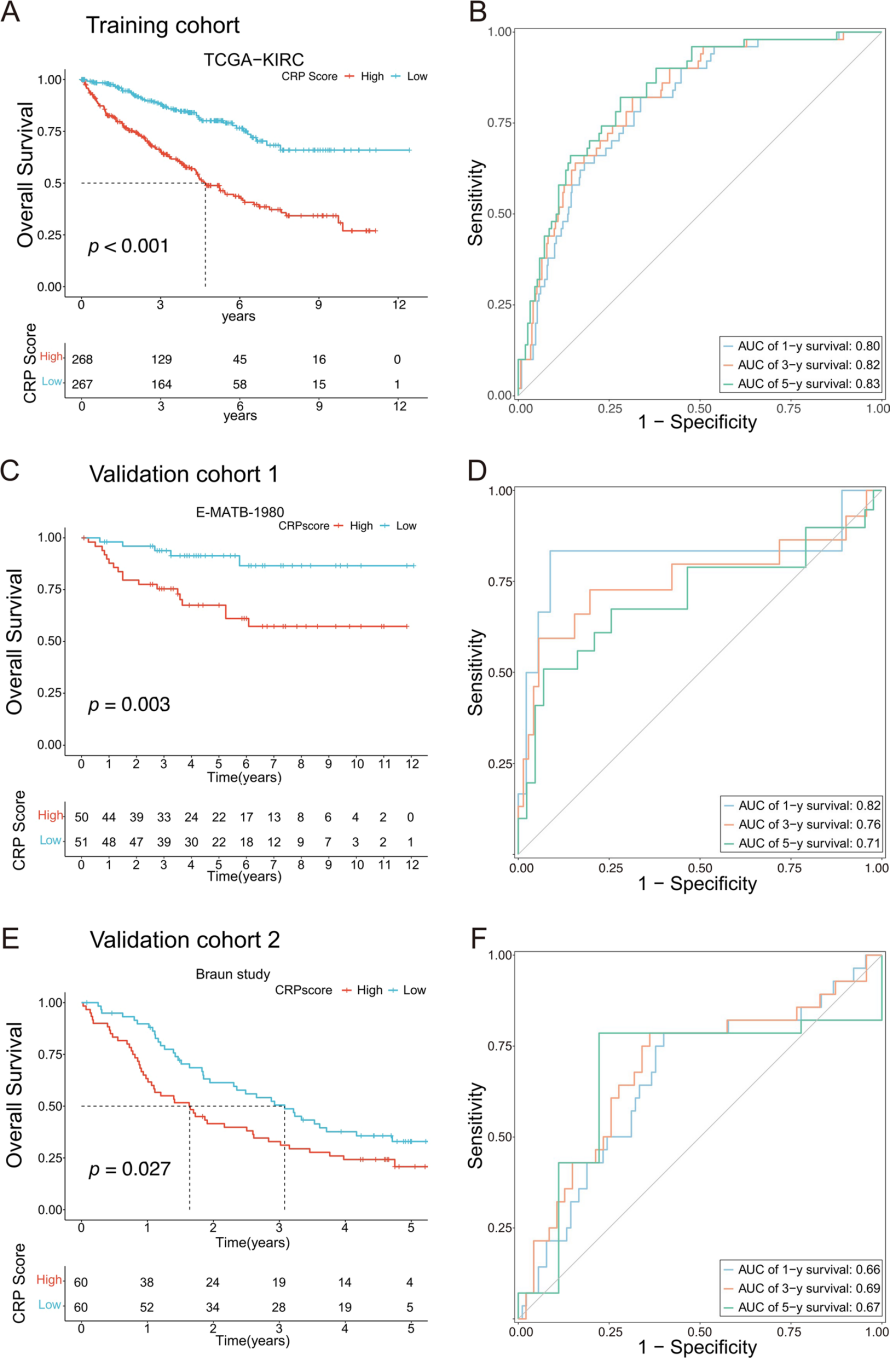
Construction and validation of CRP model in ccRCC. **A** The OS curve in the light of CRP model in TCGA-KIRC (n = 353). **B** The time-dependent receiver operating characteristic (tdROC) curve analysis of CRP model for 1,3 and 5 years. (**C**–**F**) K-M survival analysis for patients was divided into high- and low-risk CRP in the E-MTAB-1980 cohort (**C**, n = 101) and Braun cohort (**E**, n = 120); The tdROC curve (1, 3, 5 years) analysis of CRP model in E-MTAB-1980 (**D** cohort and Braun cohort (**F**)

The Cox regression method was used to establish the CRP model (coefficient value see in Additional file 25: Table S12). Firstly, as depicted in the survival plot, samples with high CRP scores correlated with significantly decreased median survival time compared to those with low CRP scores in training cohort (*p* < 0.01, Fig. 4A and Additional file 5: Fig. S5A). Next, to estimate the predictive accuracy of the CRP model, the area under the time-dependent receiver operating characteristic curve (AUC) value for OS reached 0.80 (1 year), 0.82 (3 years), and 0.83 (5 years), illustrating significantly statistical separation capability (Fig. 4B). The AUC curves evaluated the predictive effects of the CRP model of PFS (1-years: 0.72, 3-years: 0.74, 5-years: 0.76, Additional file 5: Fig. S5B). Braun's study (the patients who were without treated Everolimus in CM-025 cohort) and E-MTAB-1980 were regarded as externally validated cohorts for the CRP model. KM survival curves demonstrated the notable survival advantage for samples with lower CRP scores in the E-MTAB-1980 cohort (Fig. 4C) and the Braun’s cohort (Fig. 4E). Moreover, the AUC curves showed the extraordinary predictive effects of the CRP model for OS (1-years: 0.82, 3-years: 0.76, 5-years: 0.71) in the E-MTAB-1980 cohort (Fig. 4D), despite a slight decrease of AUC curve (1-years: 0.66, 3-years: 0.69, 5-years: 0.67) in the Braun cohort (Fig. 4F). Transcriptome characteristics with the training cohort (Additional file 6: Fig. S6A) and validation cohorts (the Braun’s cohort: Additional file 6: Fig. S6B, E-MTAB-1980 cohort: Additional file 6: Fig. S6C) are exhibited in heatmap plots. Thus, we defined the patients with high CRP scores as lower CEScore and shorter median survival times and those with low CRP scores as higher CEScore and longer median survival time in ccRCC.

### Construct and validate nomogram model based on relevant clinical information and CRP score

Next, we performed the subgroup analysis to verify the prognostic value of the CRP model in different subgroups of KIRC patients. As for Stage I-II and III-IV KIRC, a higher CRP score showed worse OS and PFS outcomes (*p* < 0.05, Additional file 7: Fig. S7A–D and Additional file 26: Table S13). Similarly, in Grade 1–2 or 3–4 KIRC samples, those in the higher CRP score group had shorter OS and PFS median survival time (*p* < 0.05, Additional file 7: Fig. S7E–H). Furthermore, OS and PFS survival results illustrated that KIRC patients with higher CRP scores had a poor prognosis (*p* < 0.05, Additional file 7: Fig. S7I–L) between younger (Age <  = 65) and older (Age > 65). When KIRC patients were separated from female and male groups, the prognostic CRP scores were consistent (*p* < 0.001, Additional file 7: Fig. S7M–P).

**Fig. 5:**
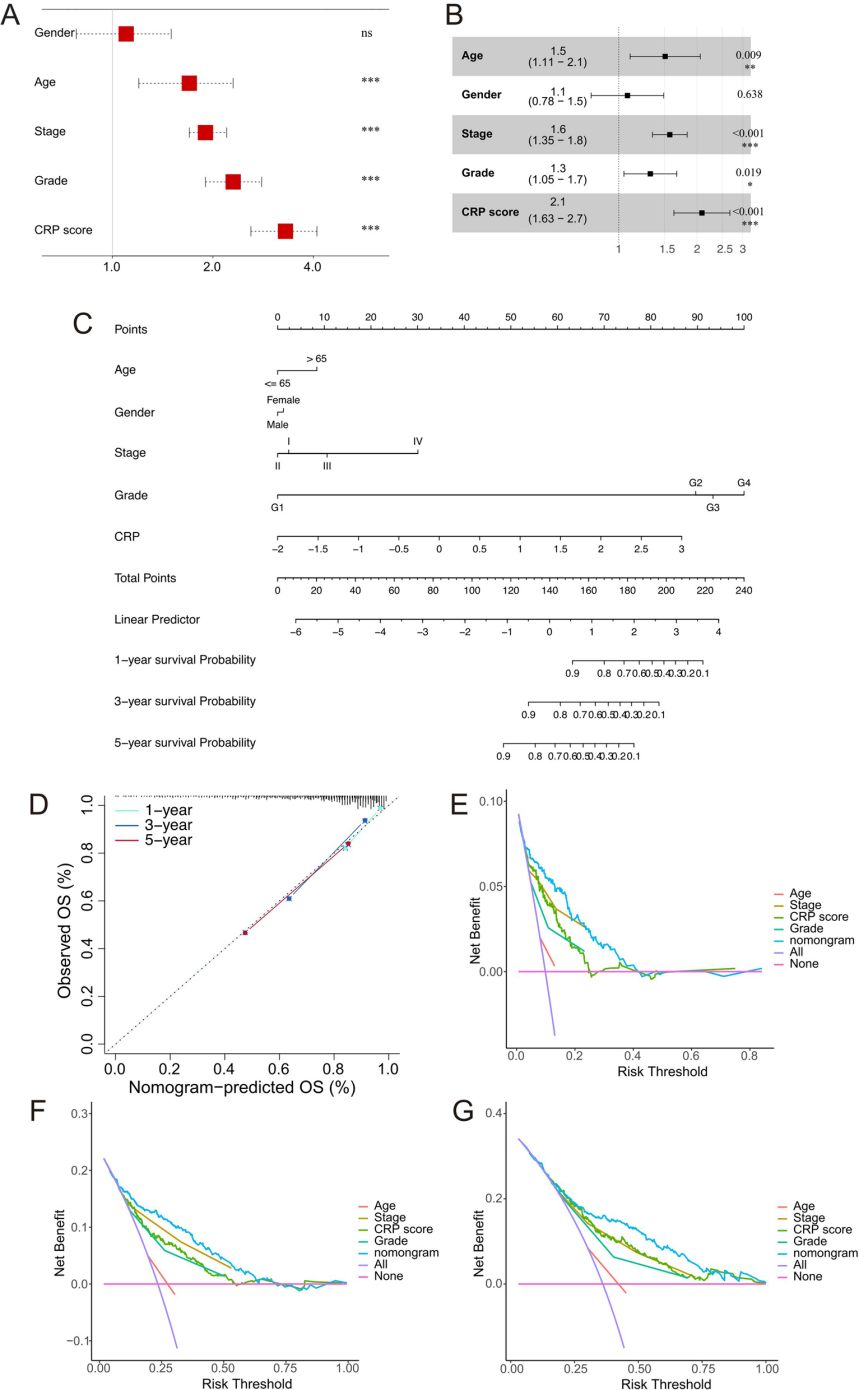
Construction and evaluation of nomograms. **A**, B The univariate (**A**) and multivariate (**B**) Cox analyses of clinicopathologic data and CRP score for OS outcomes. *p < 0.05; **p < 0.01; ***p < 0.001; ****p < 0.0001; ns: no significance. C The predictive nomogram of OS at 1-, 3-, and 5-years in TCGA-KIRC. D Calibration plots of 1-, 3-, and 5-years were utilized to evaluate the predictive accuracy of OS in the CRP model. **E**–**G** Decision curve analysis to assess the clinical utility of 1- (**E**), 3- (**F**), and 5-years (**G**) nomogram

Moreover, univariate and multivariate Cox analyses were used to assess whether the CRP model is an independent prognostic indicator for OS and PFS in KIRC (relative baseline data see in Additional file 27: Table S14). The results of univariate Cox table show that the Age, Stage, Grade, and CRP score could independently predict OS expectancy, respectively (Fig. 5A, B). In addition, the Gender, Stage, Grade, and CRP score could independently predict PFS probability (Additional file 8: Fig. S8A, B). Finally, we constructed OS and PFS nomograms to help clinicians conveniently use the CRP model in combination with the above clinical characteristics to predict the survival expectancy of a specific patient with KIRC (Fig. 5C and Additional file 8: Fig. S8C). The calibration curves and decision curve analyses implied the remarkable accuracy of OS (Fig. 5D–G) and PFS nomograms (Additional file 8: Fig. S8D–G). Our analyses indicated that the CRP model has a superior clinical benefit for KIRC patients.

### CRP score predict response to mTOR inhibitor treatment

**Fig. 6:**
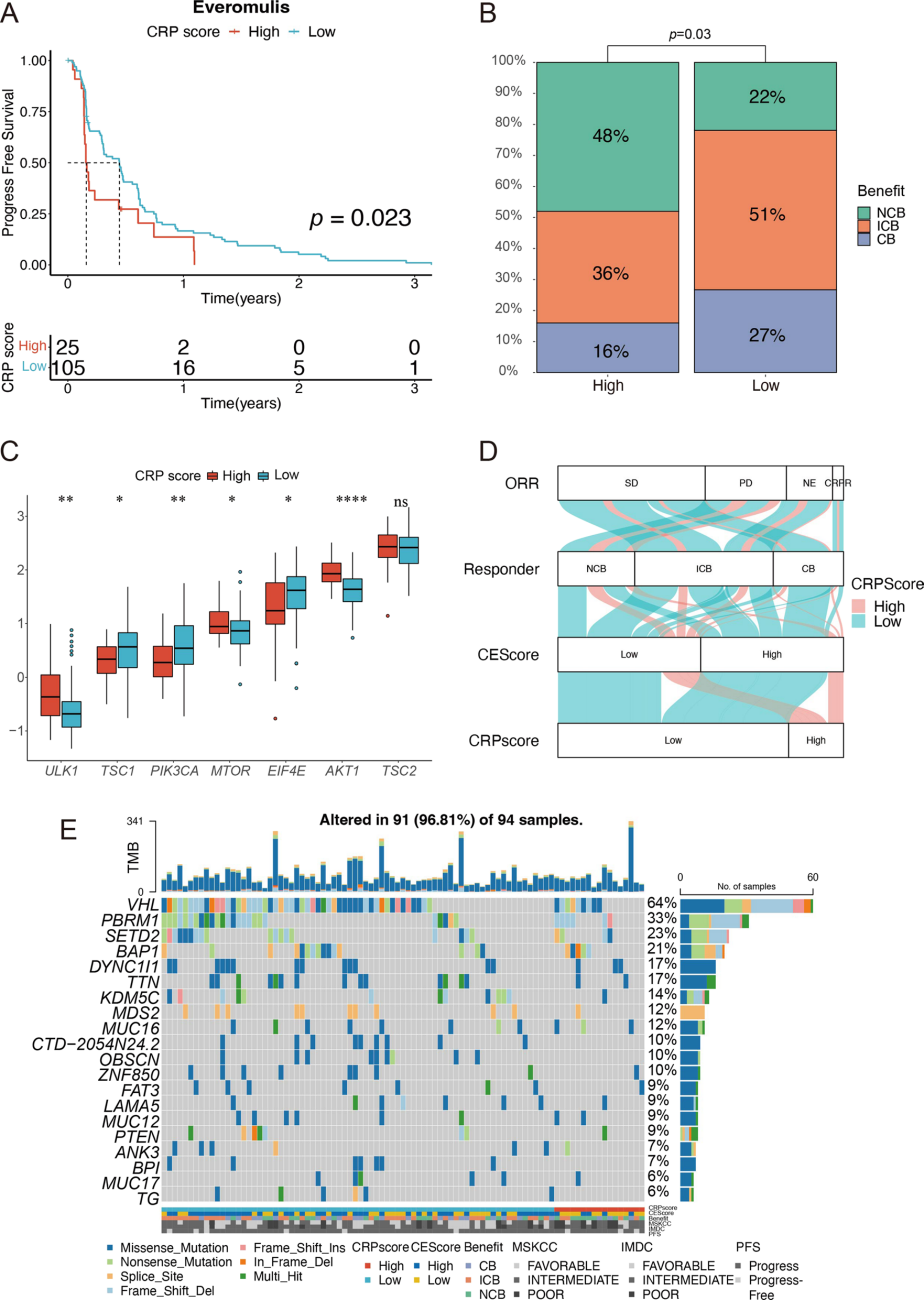
CRP model could predict the clinical benefit of Everolimus. **A** K-M survival analysis assesses the progression-free survival in the Everolimus cohort. **B** Stacked bar graphs to depict anti-Everolimus monotherapy clinical benefits from CheckMate-025; *NCB* no clinical benefit, *ICB* intermediate clinical benefit, *CB* clinical benefit. **C** Abnormally differential expression of mTOR-related genes between high- and low-risk CRP score in Everolimus cohort. *p < 0.05; **p < 0.01; ***p < 0.001; ****p < 0.0001; ns: no significance. **D** Sankey plot of treatment clinical benefit patterns between CRP score and score. **E** The oncoplot shows the overview of somatic mutations in the Everolimus-treated patients

Copper could induce autophagy via oxidative stress-dependent AMPK-mTOR pathway [35]. We investigated CRP score associated with the responder with treated mTOR inhibitor in CM-025 cohort and their survival difference. As depicted in survival curves, higher CRP score group had a shorter median survival time (*p* < 0.05, Fig. 6A). In the CM-025 cohort, the CRP score was more elevated in most patients who had undergone no clinical benefit from Everolimus than in those who intermediate clinical benefit and clinical benefit (*p* = 0.03, Fig. 6B). In addition, the CRP score can distinguish mTOR-related upstream and downstream gene expression, including *ULK1, TSC1, PIK3CA, MTOR, EIF4E*, and *AKT1* (*p* < 0.05, Fig. 6C). We also established a Sankey diagram showing the relationship between CRP score, CEScore, Responder, and their roles in the CM-025 cohort (Fig. 6D). The oncoplot depicted that VHL (Low: 48% vs. High: 36%) mutation frequencies in low CRP scores were higher than in high CRP scores, but PBRM1 (Low: 18% vs. High: 24%) in high CRP score were higher than in low CRP score (Fig. 6E).

## Discussion

Although the toxic mechanism of other crucial metals (such as iron-induced ferroptosis) is well established, the mechanisms of copper-induced cytotoxicity just elucidated its specific process [1, 36]. However, it has not been systematically described in the pan-cancer cohort.

This study demonstrated the cuproptosis features of multi-omics, including global alterations of CRGs at genetic, epigenetic, and transcriptional levels in the TCGA pan-cancer cohort (Additional file 9: Fig. S9). Our genetic analysis revealed a high frequency of copy number alterations of CRGs in HNSC, LUSC, UCEC, LUAD, BLCA, and STAD (Fig. 1A). The spearman results confirmed that CNA positively correlated with most CRGs expression, especially for cuproptosis positive genes (*PDHB, FDX1*, and *DLAT*), indicating that CNV could affect CRGs expression, in turn, contribute to tumorigenesis. Specifically, *PDHB* was frequently *Del* in LUSC and was related to worse OS in non-small cell lung cancer, which agrees with the past result [37]. We found that hypermethylation and CNV *Del*-mediated downregulation of *CDKN2A* was associated with poor survival in KIRC [38]. Numerous studies have proved that tumorigenesis of various cancers was correlated with the hypermethylation of *CDKN2A* [39–43]. Moreover, there are many CRGs without significantly differential methylation, but the expression of CRGs has a negative correlation with its inmost of cancer (Fig. 1C and Additional file 1: Fig. S1A). In addition, abnormal down-regulated expression of CRGs indicated that worse prognosis in KIRC (Fig. 1E). Thus, the above results suggest that abnormally genetic and epigenetic regulation may regulate CRG expression, which further affects the prognosis of samples in part of cancers (≥ 3 CRGs, such as BRCA, KIRC, LIHC, LGG, LUSC, MESO, and SKCM).

To further characterize the status of cuproptosis, the CEScore was established and assessed for individuals in pan-cancer. Interestingly, the distribution feature of CEScore in kidney cancer depends on their pathological differences. However, the patients with high CEScore and worse prognosis in KICH are the opposite phenomenon in KIRC and KIRP (Fig. 2C, D). Although the patients with lung cancers have lower CEScore, it showed the OS and PFS prognostic difference between LUSC and LUAD. We next exploited the association between CEScore and its significantly related pathway enrichment. The CEScore was notably associated with cancer-related signaling pathways, including oxidative phosphorylation (31/32), mTORC1 signaling (31/32), and metabolism signaling in most cancer. These enrichment results are consistent with those previously reported [1, 35, 44–46]. Guo et al. validated that copper-induced spermatogenesis dysfunction was protected by inducing autophagy via the ROS-dependent AMPK-mTOR pathway [35]. Amchandani et al. discovered that copper deficiency inhibits cancer metastasis via modulating oxidative phosphorylation, with the AMPK/mTORC1 energy sensor as a critical downstream manner [47]. On the one hand, remarkably higher levels of copper level subsequently increased lipid peroxidation and regulated copper transport to overcome drug resistance in RCC patients [5, 48]. On the other hand, the OS and PFS Cox results indicated the most significant prognostic differences of CEScore in KIRC (*p* < 0.001, Fig. 2C, D). Therefore, we established the CRP model to characterize cuproptosis-associated oxidative phosphorylation status and specific drug-resistant in KIRC. In addition, we also investigate the other significant GSEA terms (GSEA term in pan-cancer cohorts ≥ 30) and their association with prognosis in pan-cancer cohort. Noticeable, although these terms have (protein secretion: 32/32, mTORC1 signaling: 31/32, adipogenesis: 30/32) less normalized enrichment score than oxidative phosphorylation (Fig. 2E), there are significantly correlation with the prognosis of multiple cancer types (Additional file 28: Table S15 and Additional files 10–13: Fig. S10–13). The crosstalk by the other significant items besides the oxidative phosphorylation pathway with cuproptosis deserves further exploration.

We firstly got 34 CEScore-associated oxidative phosphorylation genes involved in cuproptosis and extracted a seventeen-gene signature to construct the CRP model using the reliable Cox regression method. Then, *PDK4, OGDH, ACAT1, ATP1B1, ACADSB, MPC1, BDH2, ALDH6A1, PRDX3, ATP6V1C1, AFIM1, HSPA9, DLD, SDHC*, and *SDHD* were identified as protective factors; *RHOT2* and *COX5B* were regarded as risk factors. According to the CRP model, the patients with high CRP scores and lower CEScore indicated a worse prognosis in the training cohort (TCGA-KIRC) and validated cohort (E-MTAB-1980, CM-025). To estimate the reliability in prediction, we calculated the CRP model of AUC in 1-,3-, and 5-years and further validated the accuracy of the nomogram model based on the CRP model.

Based on the significantly high enrichment score (ES) value of CEScore in mTOR-related pathways, we found that low-risk CRP patients treated with Everolimus (mTOR inhibitor) may be acquired better clinical efficiency. Guo et al. have reported that cupric ion contributes to autophagy via oxidative stress-dependent AMPK-mTOR pathway in mouse spermatogenic cells [35]. Furthermore, there is no study between mTOR inhibitor and cuproptosis in cancer. Two significant copper therapeutic strategies have been applied to copper dysregulated, including copper chelation, copper ionophores, and inhibitor. For example, Tetrathiomolybdate, a copper chelation, has been revealed to be stable, depletes copper, and is well-tolerated in phase II trials of advanced kidney cancer [21]. Preclinical studies have demonstrated that limiting the availability of copper dependence is an effective strategy for blocking KRAS-driven and autophagy-dependent tumor growth and survival in copper dysregulated diseases [49, 50]. A phase II study (NCT03034135) recently showed that adding Cu–DSF to patients with temozolomide-resistant glioblastoma is well tolerated [19]. Taken together, the novel named cuproptosis in a subset of cancer has sufficient potential in therapeutic interventions.

Although we tried to infer the cuproptosis status exactly, there could be a variety of flaws because existing omics data only acquire RNA-seq quantifications for CRGs, but the cuproptosis process relies on proteins. Furthermore, the precise molecular pathways and mechanisms behind cuproptosis remain elucidated, limiting CEScores’ sensitivity and specificity. Although the CRP model could characterize cuproptosis status and the clinical benefit of mTOR inhibitor, there are still more limitations in invalidation and application.

## Conclusions

This study clarified the landscape of multi-omics features for cuproptosis and constructed CEScore to characterize cuproptosis status in pan-cancer. We were providing a precise and stable CRP model for predicting cuproptosis-associated survival prognosis and mTOR-targeted therapies in ccRCC. Our study provides a rationale for copper-induced death-specific tumor model selection and novel therapies targeting cuproptosis therapy.

## Supplementary Information

**Additional file 1: Fig. S1.** Multi-omics analysis of CRGs. (A) Differential methylation between tumor and normal samples of CRGs in pan-cancer; FDR count: the significance of FDR; FDR: adjust p-value; Methy.diff(T-N): differential methylation (Tumor vs. Normal) (B) Co-occurrence and mutually exclusive of CRGs in pan-cancer.

**Additional file 2: Fig. S2.** CRGs expression analysis. (A) The paired tumor and normal samples were used to identify differential expression of CRGs in pan-cancer (14/32). (B) The subtype relevant changes of CRGs expression in pan-cancer; FDR: False discovery rate; FDR count: the significance of FDR.

**Additional file 3: Fig. S3.** The OS rate between high-CEScore and Low-CEScore in pan-cancer.

**Additional file 4: Fig. S4.** The PFS rate between high-CEScore and Low-CEScore in pan-cancer.

**Additional file 5: Fig. S5.** The PFS KM survival (A) and ROC (B) curves in the light of the CRP model in TCGA-KIRC (n = 353).

**Additional file 6: Fig. S6.** Heatmap shows the transcriptome characteristics of 17 risk genes between high- and low-CRP scores in TCGA-KIRC (A, n = 353), E-MTAB-1980 cohort (B, n = 101), Braun cohort (C, n = 120).

**Additional file 7: Fig. S7.** Sub-group Survival analysis of KIRC. (A-P) The survival curve plot shows differences in OS/PFS outcomes between high- and low-CRP scores in different clinical subgroups, including Stage (I-II: A and C, III-IV: B and D), Grade (I-II: E and G, III-IV: F and H), age (< = 65: I and k, > 65: j and L), and Gender (Male: m and o, Female: N and P).

**Additional file 8: Fig. S8.** Construction and evaluation of nomograms for PFS. (A-B) The univariate (A) and multivariate (B) Cox analyses of clinicopathologic data and CRP score for PFS outcomes. *p < 0.05; **p < 0.01; ***p < 0.001; ****p < 0.0001; ns: no significance. (C) The predictive nomogram of PFS at 1-, 3-, and 5-years in TCGA-KIRC. (D) Calibration plots of 1-, 3-, and 5-years were utilized to evaluate the predictive accuracy of PFS in the CRP model. (E–G) Decision curve analysis to assess the clinical utility of 1- (E), 3- (F), and 5-years (G) nomogram.

**Additional file 9: Fig. S9.** The flowchart of this study.

**Additional file 10: Fig. S10.** The association between the protein secretion and prognosis in pan-cancer cohort.

**Additional file 11: Fig. S11.** The correlation between the oxidative phosphorylation and prognosis in pan-cancer cohort.

**Additional file 12: Fig. S12.** The association between the adipogenesis term and prognosis in pan-cancer cohort.

**Additional file 13: Fig. S13.** The relation between the mTORC1 signaling term and prognosis in pan-cancer cohort.

**Additional file 14: Table S1.** Cuproptosis-regulated genes list.

**Additional file 15: Table S2.** The somatic mutation of CRGs in pan-cancer.

**Additional file 16: Table S3.** The somatic copy number alteration of CRGs in pan-cancer.

**Additional file 17: Table S4.** Differentially expressed CRGs in pan-cancer.

**Additional file 18: Table S5.** Subtype distribution of CRGs in multiple cancers.

**Additional file 19: Table S6.** CEScore in pan-cancer.

**Additional file 20: Table S7.** Cox analysis of CEScore in pan-cancer.

**Additional file 21: Table S8.** Clinical baseline data of CEScore in pan-cancer.

**Additional file 22: Table S9.** Pathway enrichment of CEScore in pan-cancer.

**Additional file 23: Table S10.** OncoSore and oncoScape of CEScore-related genes.

**Additional file 24: Table S11.** Univariate Cox analysis of cuproptosis-related targeted genes in KIRC.

**Additional file 25: Table S12.** CRP genes and each coefficient.

**Additional file 26: Table S13.** Univariate Cox analysis in clinical data in KIRC.

**Additional file 27: Table S14.** Clinical baseline data of training cohort and validate cohorts in ccRCC.

**Additional file 28: Table S15.** The expression and prognostic analysis of the other significant GSEA terms.

## Data Availability

The gene expression, clinical-associated data, and somatic mutation data are available in the Xena database (https://xenabrowser.net/datapages/) and cBioPortal website (https://www.cbioportal.org/); the E-MTAB-1980 cohort of RNA-seq and clinical metadata are downloaded at the website (https://www.ebi.ac.uk/arrayexpress/experiments/E-MTAB-1980/); the CheckMate 025 data were extracted from Braun study (https://www.ncbi.nlm.nih.gov/pmc/articles/PMC7499153/).

## Abbreviations

ACC: Adrenocortical carcinoma
BLCA: Bladder urothelial carcinoma
BRCA: Breast invasive carcinoma
CESC: Cervical squamous cell carcinoma and endocervical adenocarcinoma
CHOL: Cholangiocarcinoma
COAD: Colon adenocarcinoma
DLBC: Lymphoid neoplasm diffuse large b-cell lymphoma
ESCA: Esophageal carcinoma
GBM: Glioblastoma multiforme
HNSC: Head and neck squamous cell carcinoma
KICH: Kidney chromophobe
KIRC: Kidney renal clear cell carcinoma
KIRP: Kidney renal papillary cell carcinoma
LGG: Brain lower grade glioma
LIHC: Liver hepatocellular carcinoma
LUAD: Lung adenocarcinoma
LUSC: Lung squamous cell carcinoma
MESO: Mesothelioma
OV: Ovarian serous cystadenocarcinoma
PAAD: Pancreatic adenocarcinoma
PCPG: Pheochromocytoma and paraganglioma
PRAD: Prostate adenocarcinoma
READ: Rectum adenocarcinoma
SARC: Sarcoma
SKCM: Skin cutaneous melanoma
STAD: Stomach adenocarcinoma
TGCT: Testicular germ cell tumors
THYM: Thymoma
THCA: Thyroid carcinoma
UCS: Uterine carcinosarcoma
UCEC: Uterine corpus endometrial carcinoma
UVM: Uveal melanoma
CRGs: Cuproptosis-regulated genes
CEScore: Copper enrichment score
CRP: Cuproptosis-related prognosis
CAN: Copy number alterations
DLAT: Dihydrolipoamide S-acetyltransferase
FDX1: Ferredoxin 1
LIAS: Lipoic acid synthetase
LIPT1: Lipoyltransferase 1
DLD: Dihydrolipoamide dehydrogenase
PDHA1: Pyruvate dehydrogenase E1 subunit alpha 1
PDHB: Pyruvate dehydrogenase E1 subunit beta
MTF1: Metal regulatory transcription factor 1
GLS: Glutaminase
CDKN2A: Cyclin dependent kinase inhibitor 2A
ATP7A: ATPase copper transporting alpha
ATP7B: ATPase copper transporting beta
SLC31A1: Solute carrier family 31 member 1
DLST: Dihydrolipoamide S-succinyltransferase
DBT: Dihydrolipoamide branched chain transacylase E2
GCSH: Glycine cleavage system protein H
LIPT2: Lipoyl (octanoyl) transferase 2
OS: Overall survival
PFS: Progress free survival
