# Supplementary figures and images for: Pan-cancer analysis of cuproptosis regulation patterns and identification of mTOR-target responder in clear cell renal cell carcinoma
Source: Biol Direct. 2022 Oct 8;17:28. doi: 10.1186/s13062-022-00340-y (PMC9548146; doi:10.1186/s13062-022-00340-y)

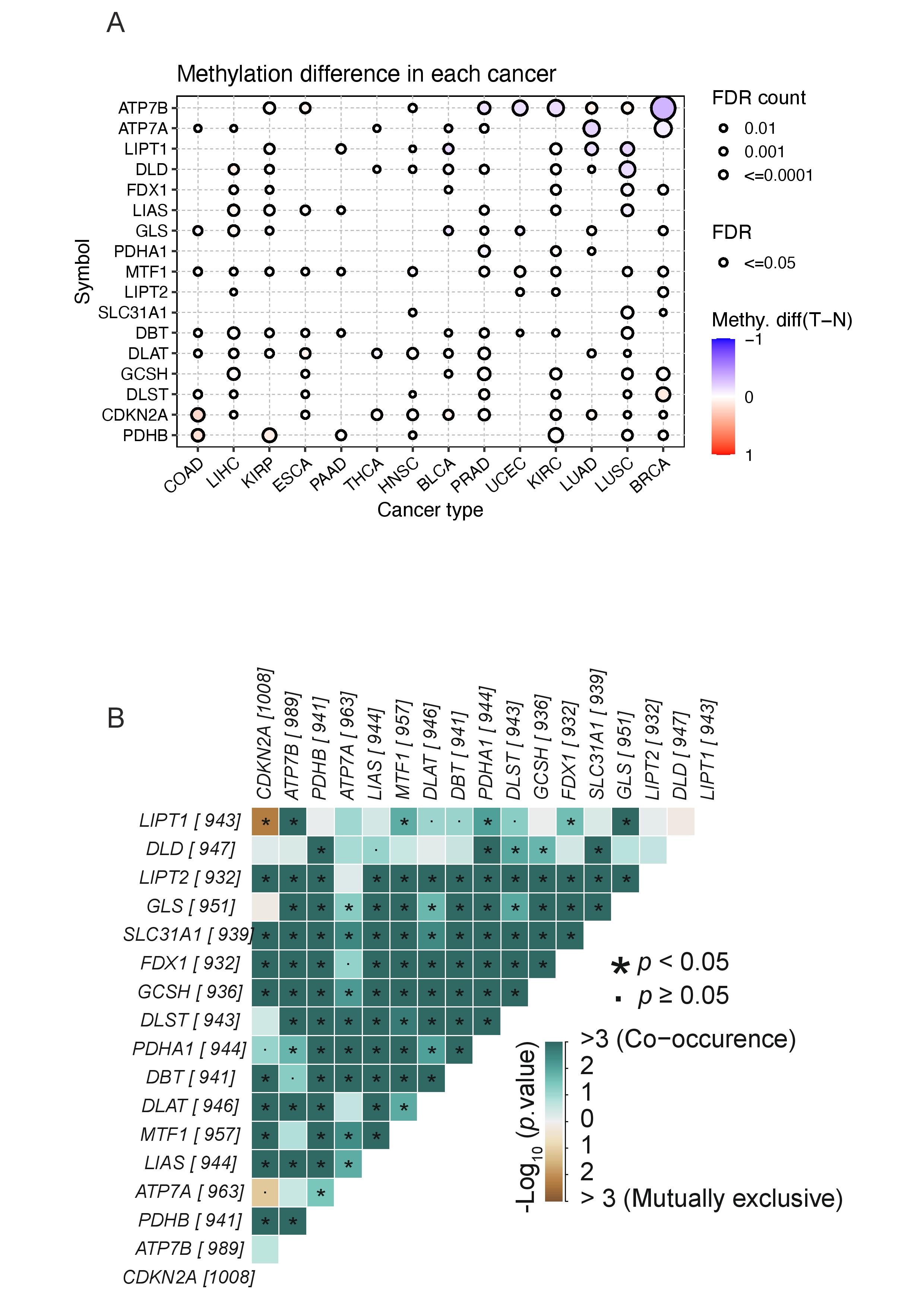

Supplement: Supplementary file 1 — Additional file 1: Fig. S1. Multi-omics analysis of CRGs. (A) Differential methylation between tumor and normal samples of CRGs in pan-cancer; FDR count: the significance of FDR; FDR: adjust p-value; Methy.diff(T-N): differential methylation (Tumor vs. Normal) (B) Co-occurrence and mutually exclusive of CRGs in pan-cancer. [file 13062_2022_340_MOESM1_ESM.jpg]

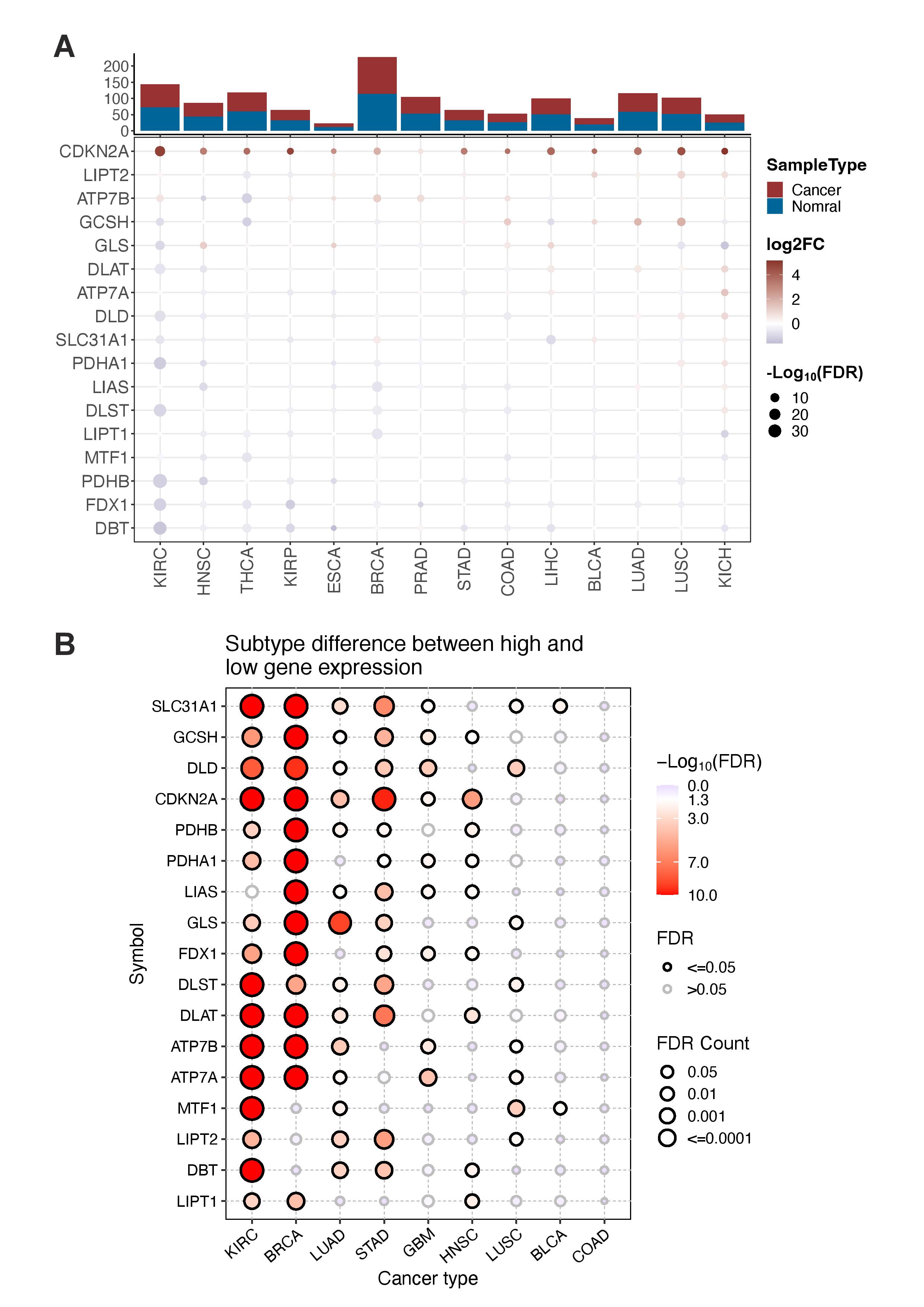

Supplement: Supplementary file 2 — Additional file 2: Fig. S2. CRGs expression analysis. (A) The paired tumor and normal samples were used to identify differential expression of CRGs in pan-cancer (14/32). (B) The subtype relevant changes of CRGs expression in pan-cancer; FDR: False discovery rate; FDR count: the significance of FDR. [file 13062_2022_340_MOESM2_ESM.jpg]

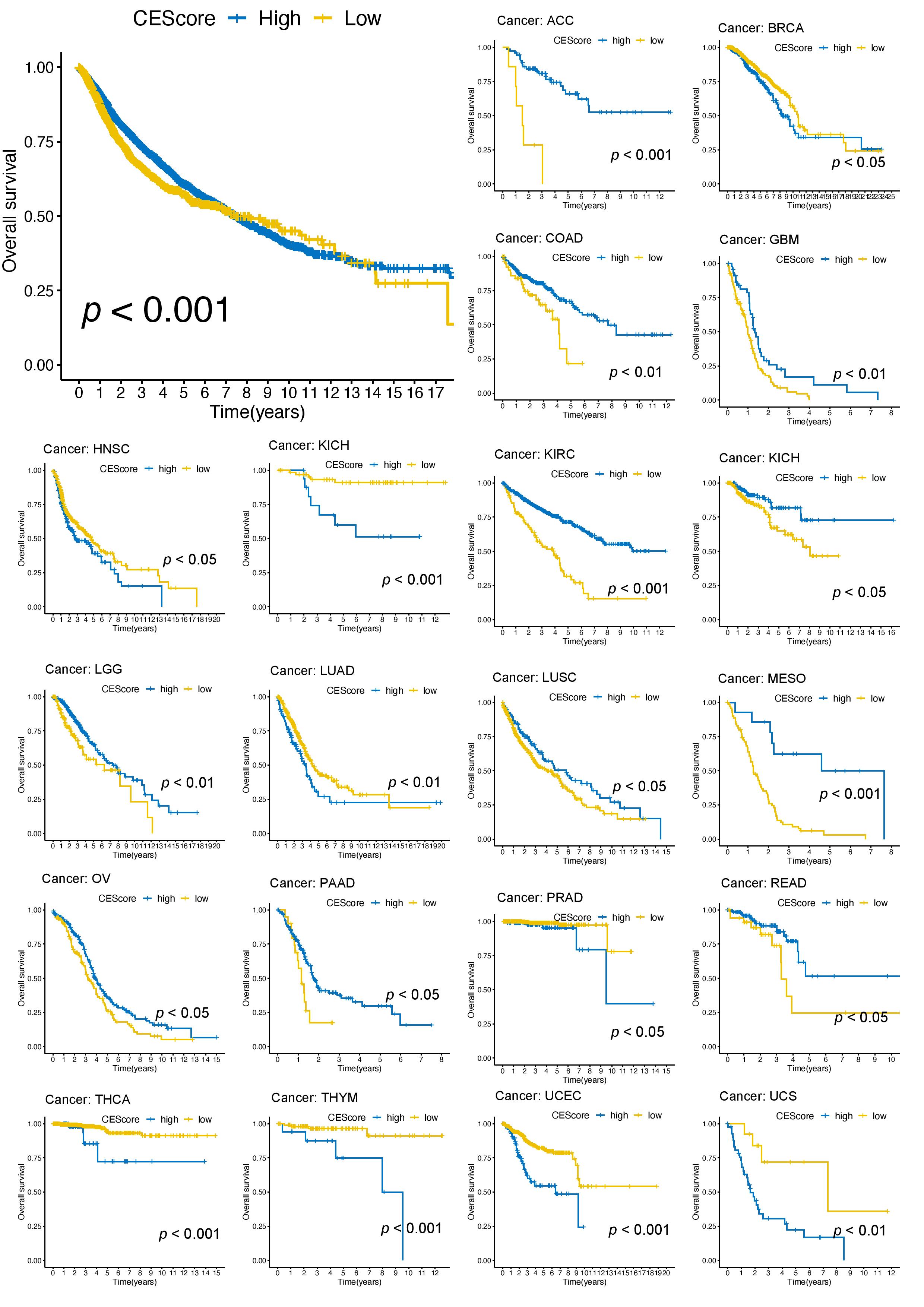

Supplement: Supplementary file 3 — Additional file 3: Fig. S3. The OS rate between high-CEScore and Low-CEScore in pan-cancer. [file 13062_2022_340_MOESM3_ESM.jpg]

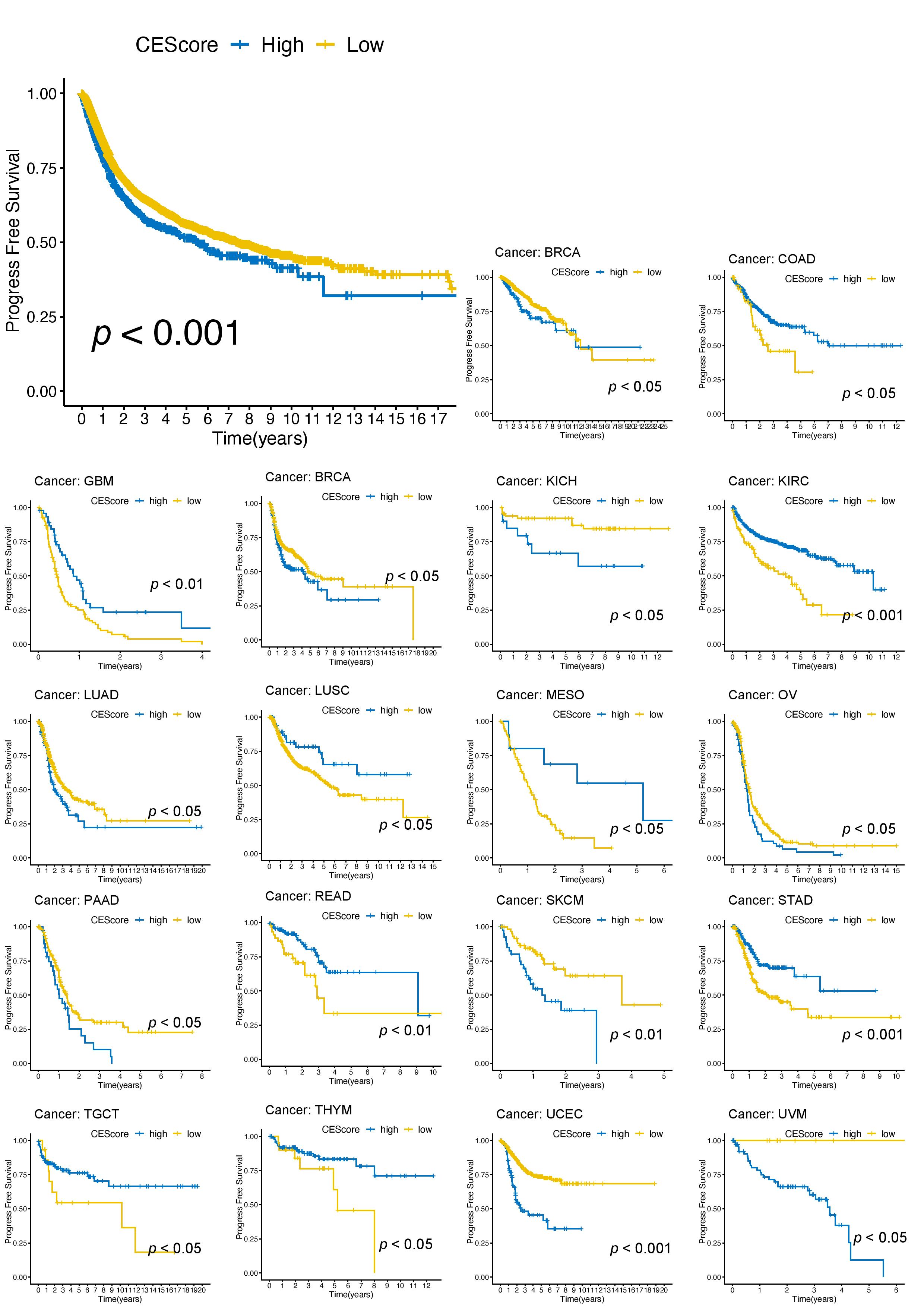

Supplement: Supplementary file 4 — Additional file 4: Fig. S4. The PFS rate between high-CEScore and Low-CEScore in pan-cancer. [file 13062_2022_340_MOESM4_ESM.jpg]

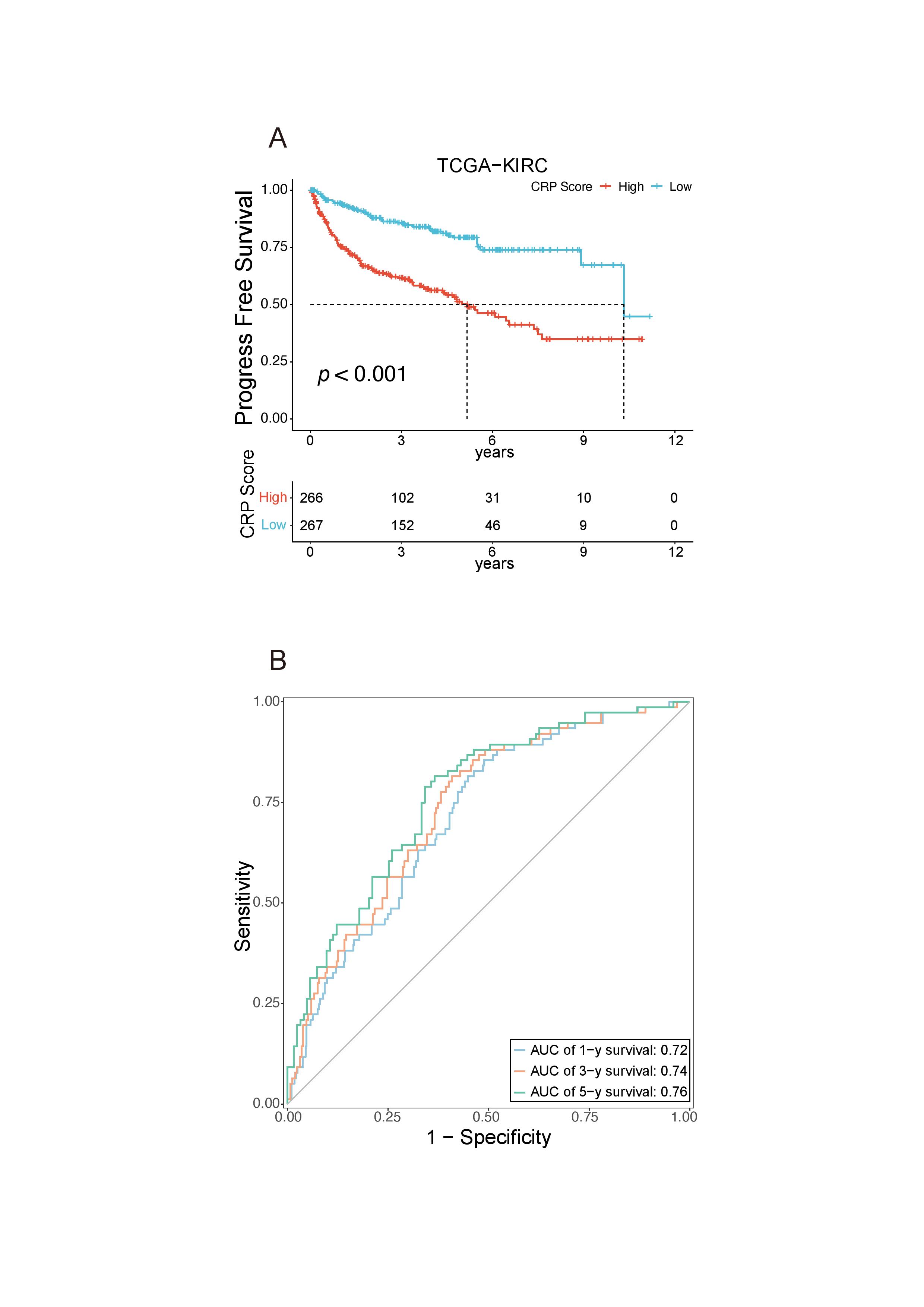

Supplement: Supplementary file 5 — Additional file 5: Fig. S5. The PFS KM survival (A) and ROC (B) curves in the light of the CRP model in TCGA-KIRC (n = 353). [file 13062_2022_340_MOESM5_ESM.jpg]

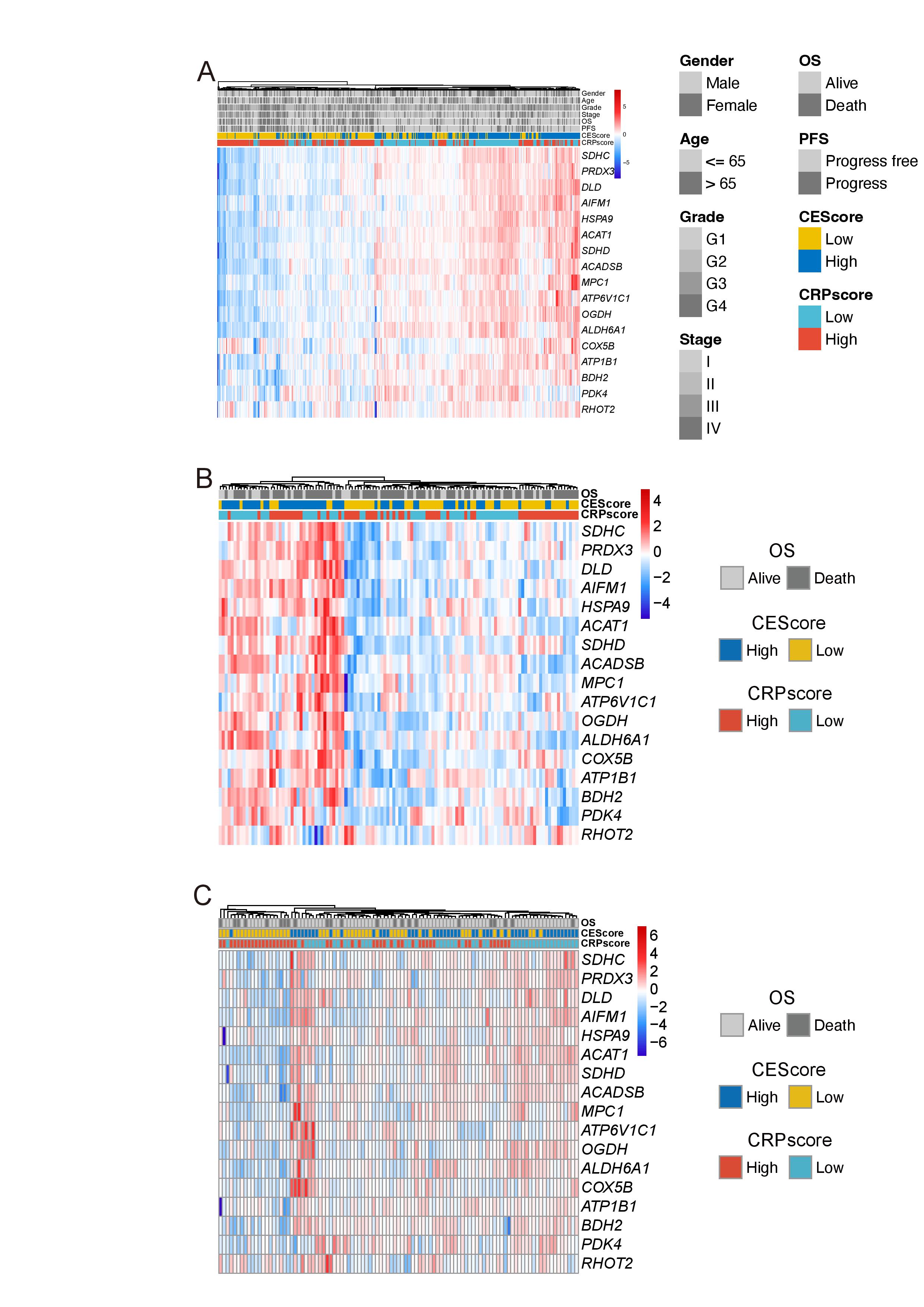

Supplement: Supplementary file 6 — Additional file 6: Fig. S6. Heatmap shows the transcriptome characteristics of 17 risk genes between high- and low-CRP scores in TCGA-KIRC (A, n = 353), E-MTAB-1980 cohort (B, n = 101), Braun cohort (C, n = 120). [file 13062_2022_340_MOESM6_ESM.jpg]

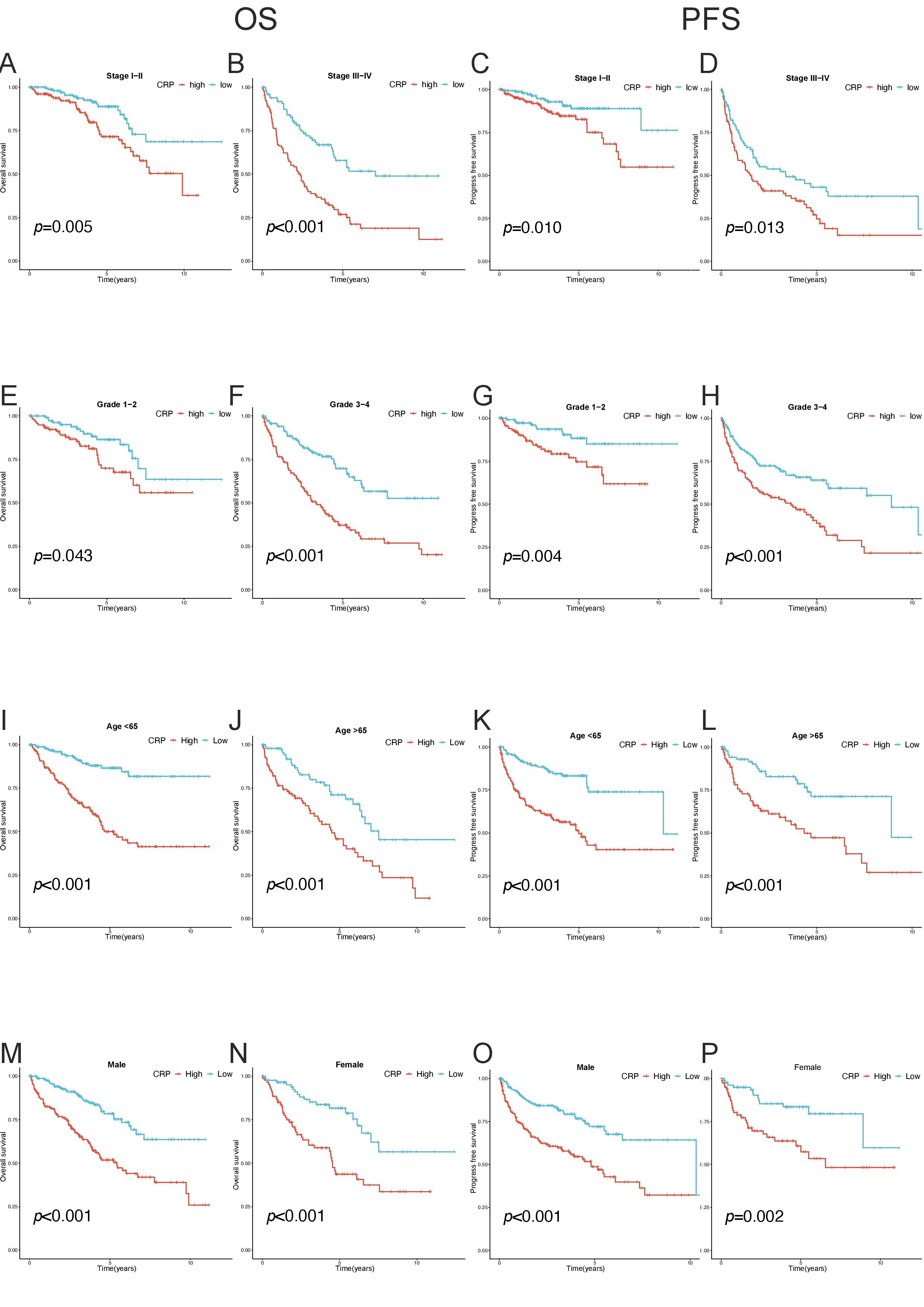

Supplement: Supplementary file 7 — Additional file 7: Fig. S7. Sub-group Survival analysis of KIRC. (A-P) The survival curve plot shows differences in OS/PFS outcomes between high- and low-CRP scores in different clinical subgroups, including Stage (I-II: A and C, III-IV: B and D), Grade (I-II: E and G, III-IV: F and H), age (< = 65: I and k, > 65: j and L), and Gender (Male: m and o, Female: N and P). [file 13062_2022_340_MOESM7_ESM.jpg]

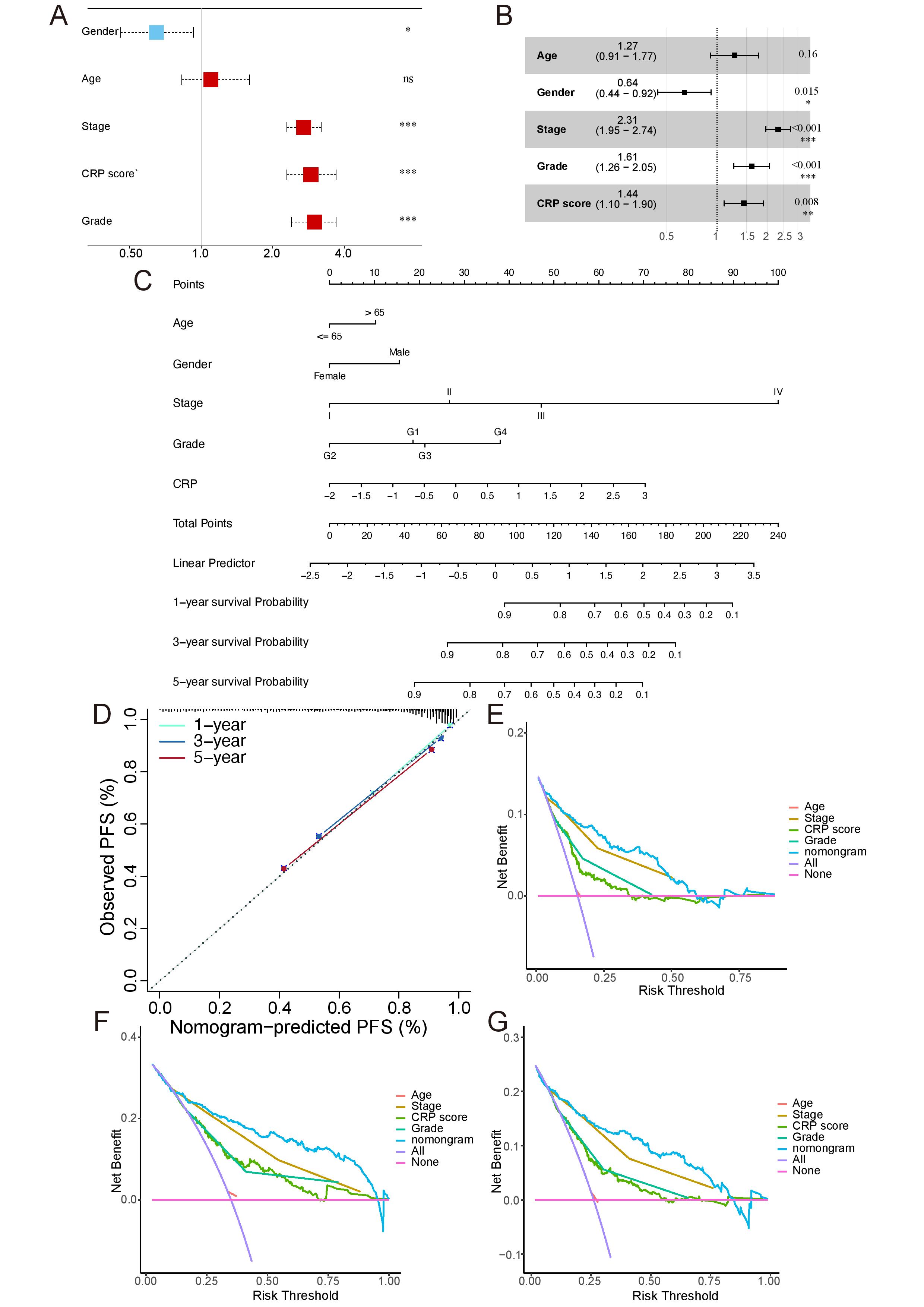

Supplement: Supplementary file 8 — Additional file 8: Fig. S8. Construction and evaluation of nomograms for PFS. (A-B) The univariate (A) and multivariate (B) Cox analyses of clinicopathologic data and CRP score for PFS outcomes. *p < 0.05; **p < 0.01; ***p < 0.001; ****p < 0.0001; ns: no significance. (C) The predictive nomogram of PFS at 1-, 3-, and 5-years in TCGA-KIRC. (D) Calibration plots of 1-, 3-, and 5-years were utilized to evaluate the predictive accuracy of PFS in the CRP model. (E–G) Decision curve analysis to assess the clinical utility of 1- (E), 3- (F), and 5-years (G) nomogram. [file 13062_2022_340_MOESM8_ESM.jpg]

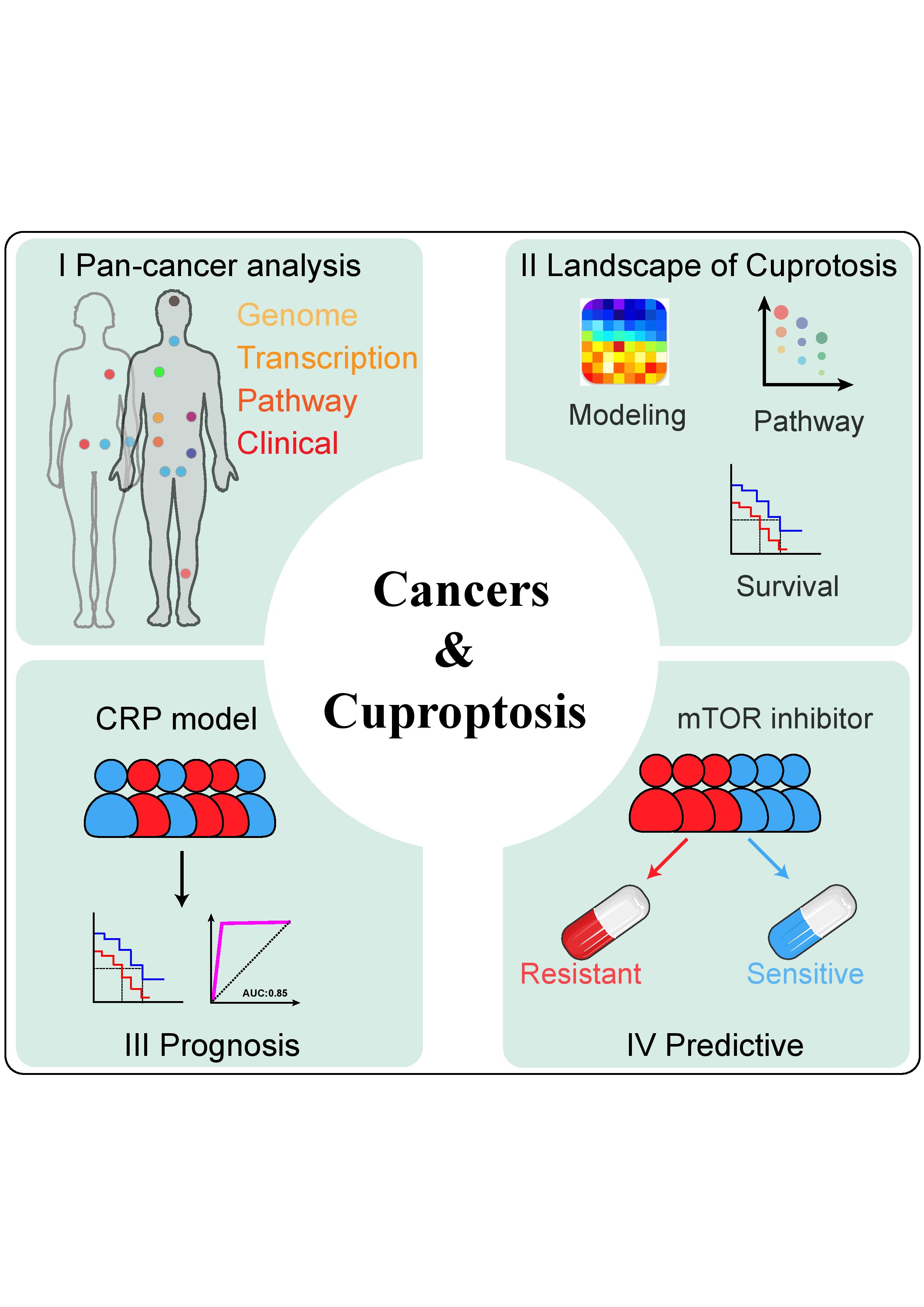

Supplement: Supplementary file 9 — Additional file 9: Fig. S9. The flowchart of this study. [file 13062_2022_340_MOESM9_ESM.jpg]

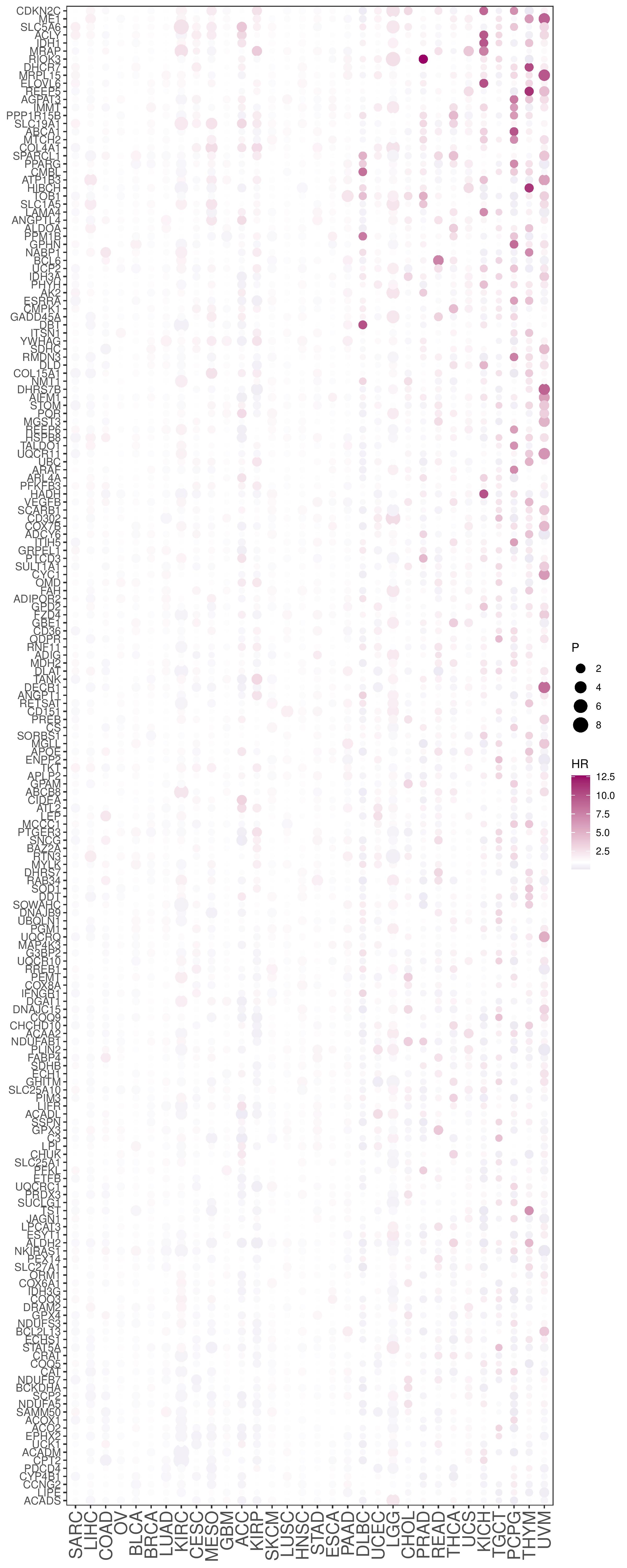

Supplement: Supplementary file 10 — Additional file 10: Fig. S10. The association between the protein secretion and prognosis in pan-cancer cohort. [file 13062_2022_340_MOESM10_ESM.jpg]

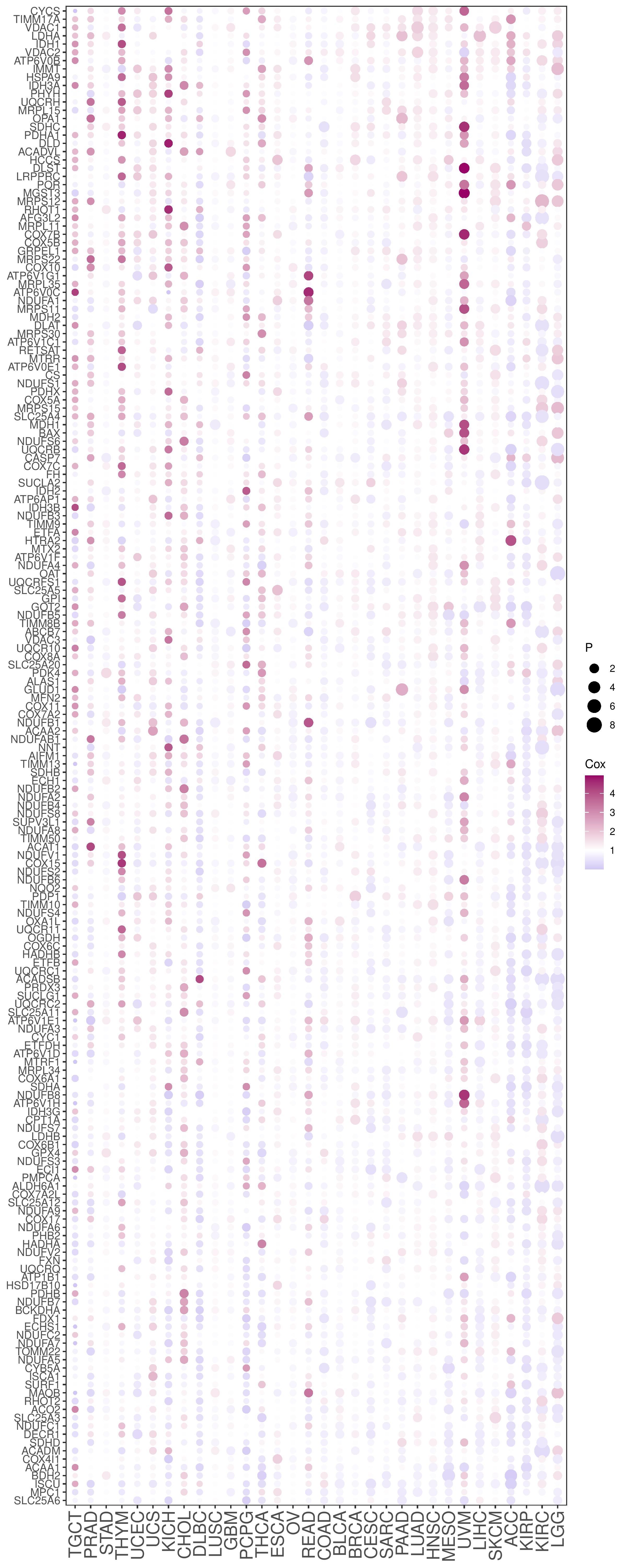

Supplement: Supplementary file 11 — Additional file 11: Fig. S11. The correlation between the oxidative phosphorylation and prognosis in pan-cancer cohort. [file 13062_2022_340_MOESM11_ESM.jpg]

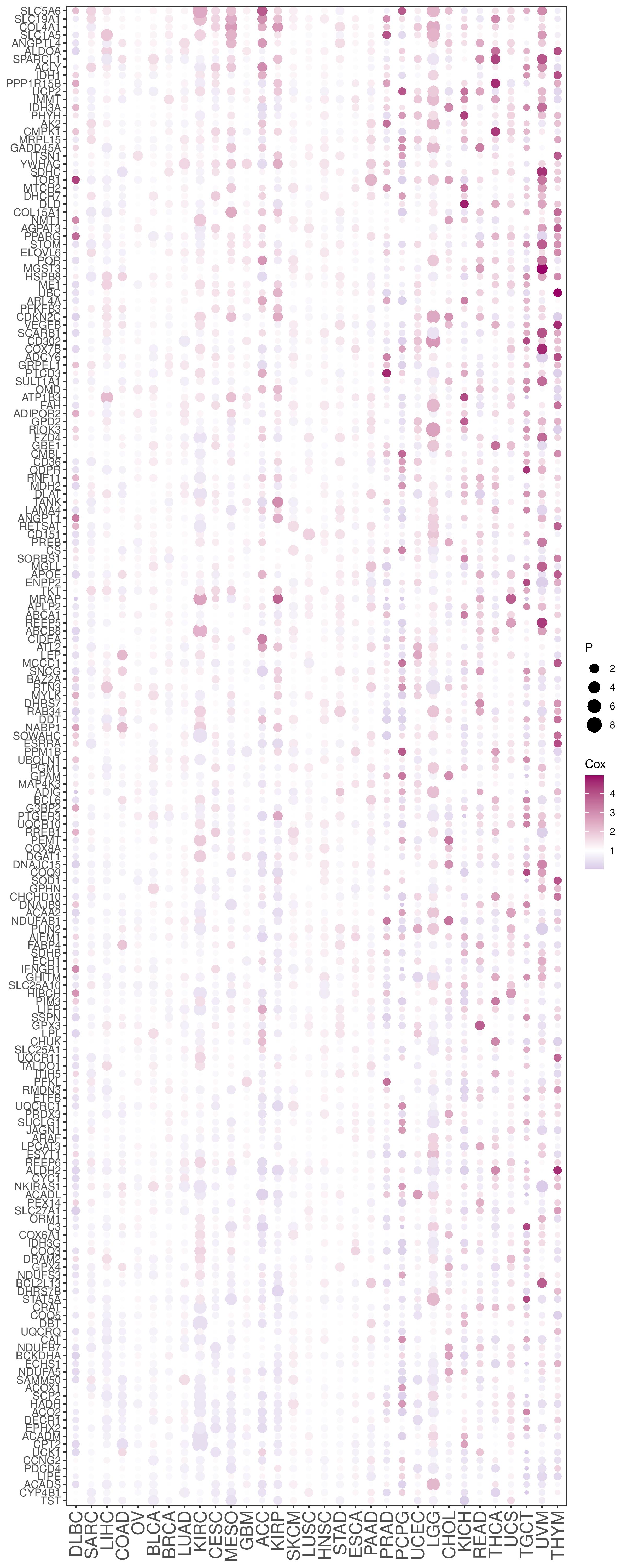

Supplement: Supplementary file 12 — Additional file 12: Fig. S12. The association between the adipogenesis term and prognosis in pan-cancer cohort. [file 13062_2022_340_MOESM12_ESM.jpg]

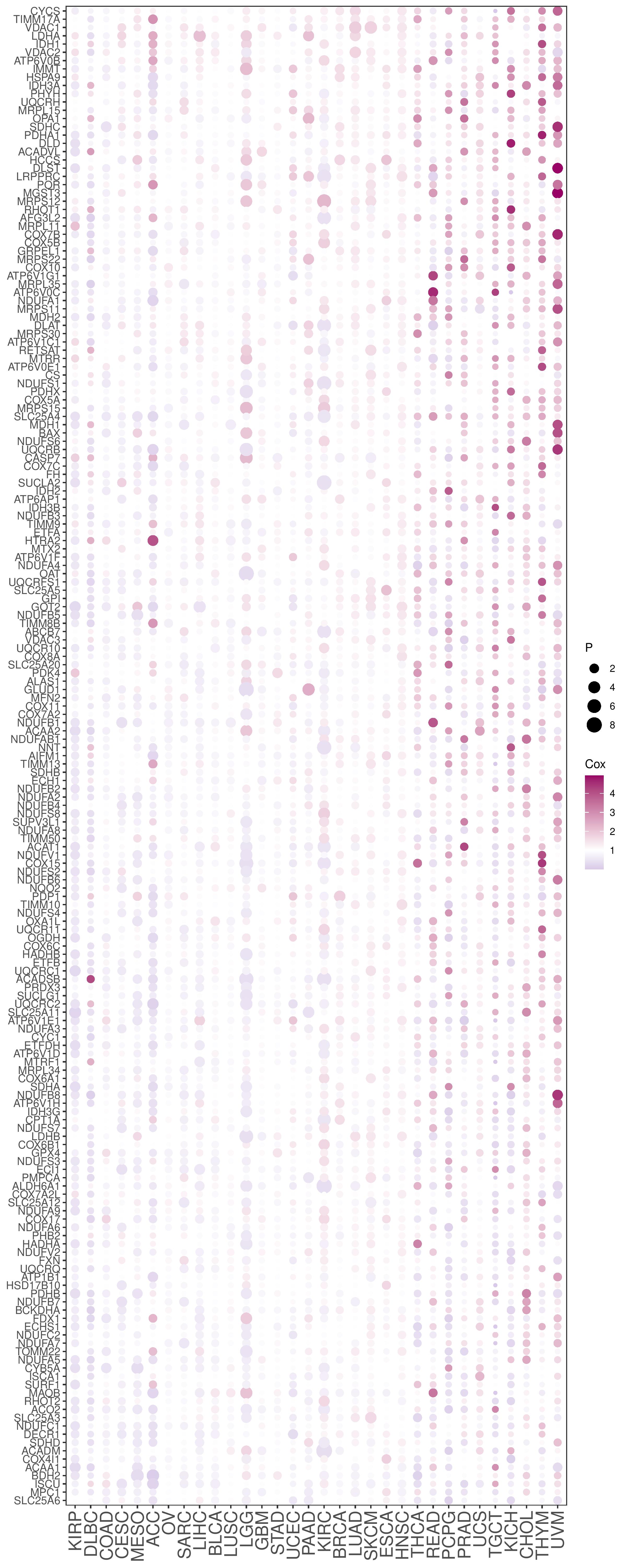

Supplement: Supplementary file 13 — Additional file 13: Fig. S13. The relation between the mTORC1 signaling term and prognosis in pan-cancer cohort. [file 13062_2022_340_MOESM13_ESM.jpg]
